# Systems perspectives on erythromycin biosynthesis by comparative genomic and transcriptomic analyses of *S. erythraea* E3 and NRRL23338 strains

**DOI:** 10.1186/1471-2164-14-523

**Published:** 2013-07-31

**Authors:** Yuan-Yuan Li, Xiao Chang, Wen-Bang Yu, Hao Li, Zhi-Qiang Ye, Hui Yu, Bao-Hong Liu, Yan Zhang, Si-Liang Zhang, Bang-Ce Ye, Yi-Xue Li

**Affiliations:** 1 Shanghai Center for Bioinformation Technology, 1278 Keyuan Road, Shanghai 201203, China; 2 Lab of Biosystems and Microanalysis, State Key Laboratory of Bioreactor Engineering, East China University of Science & Technology, Shanghai 200237, China; 3 Bioinformatics Center, Key Lab of Systems Biology, Shanghai Institutes for Biological Sciences, Chinese Academy of Sciences, 320 Yueyang Road, Shanghai 200031, China; 4 Laboratory of Chemical Genomics, School of Chemical Biology and Biotechnology, Peking University Shenzhen Graduate School, Shenzhen, 518055, China; 5 The Center for Applied Genomics, Abramson Research Center, The Children’s Hospital of Philadelphia, Philadelphia, PA 19104, USA

**Keywords:** *S. erythraea*, Erythromycin biosynthesis, Functional comparative genetics, Regulation mechanism

## Abstract

**Background:**

*S. erythraea* is a Gram-positive filamentous bacterium used for the industrial-scale production of erythromycin A which is of high clinical importance. In this work, we sequenced the whole genome of a high-producing strain (E3) obtained by random mutagenesis and screening from the wild-type strain NRRL23338, and examined time-series expression profiles of both E3 and NRRL23338. Based on the genomic data and transcriptpmic data of these two strains, we carried out comparative analysis of high-producing strain and wild-type strain at both the genomic level and the transcriptomic level.

**Results:**

We observed a large number of genetic variants including 60 insertions, 46 deletions and 584 single nucleotide variations (SNV) in E3 in comparison with NRRL23338, and the analysis of time series transcriptomic data indicated that the genes involved in erythromycin biosynthesis and feeder pathways were significantly up-regulated during the 60 hours time-course. According to our data, BldD, a previously identified *ery* cluster regulator, did not show any positive correlations with the expression of *ery* cluster, suggesting the existence of alternative regulation mechanisms of erythromycin synthesis in *S. erythraea*. Several potential regulators were then proposed by integration analysis of genomic and transcriptomic data.

**Conclusion:**

This is a demonstration of the functional comparative genomics between an industrial *S. erythraea* strain and the wild-type strain. These findings help to understand the global regulation mechanisms of erythromycin biosynthesis in *S. erythraea*, providing useful clues for genetic and metabolic engineering in the future.

## Background

*Saccharopolyspora erythraea,* formerly identified as *Streptomyces erythraeus*, is a Gram-positive filamentous bacterium. It has been used for the industrial-scale production of erythromycin A, a broad-spectrum macrolide antibiotic against pathogenic Gram-positive bacteria [1]. The antimicrobial spectrum of erythromycin A is similar to that of penicillin, and Erythromycin A is often prescribed as an alternative for patients with an allergy to penicillin. Moreover, a series of derivatives of erythromycin, derived by chemical and biotechnological transformation, have been shown to have antiparasitic, antineoplastic, immunosuppressant, neurotrophic, anti-inflammatory, and gastroenteric therapeutic activities [2]. In view of the high clinical importance of erythromycin and its derivatives, extensive efforts have been devoted to increase the erythromycin production in *S. erythraea* which has even been studied as a model system for antibiotic production [3,4].

Over the past 50 years, the *S. erythraea* strain improvement has been carried out mainly by multiple rounds of random mutagenesis and selection [2,5,6]. Meanwhile, erythromycin productivity has also been enhanced by the optimization of fermentation process [7,8]. Since 1990s, genetic studies have provided insights into the genes involved in erythromycin biosynthesis, and the erythromycin gene cluster was found to contain 20 genes arranged in four major polycistronic units [9-11], which facilitated strain improvements by genetic and metabolic engineering [12-15]. Recently, *BldD*, a key developmental regulator in actinomycetes [16,17], was identified to positively regulate the synthesis of erythromycin at the transcriptional level [18], which opened the possibility of enhancing the erythromycin production by modifying the regulation mechanism. However, *bldD* was later reported to exhibit an opposite gene expression pattern with respect to most of *ery* genes in a higher-producing strain [6], which made the regulation mechanism underlying erythromycin production complicated and confusing. As a result, the engineering works have still been focusing on erythromycin biosynthesis pathway [19] and feeder pathway [15,20].

Noticing that genetic modifications targeting to crucial genes are sometimes lethal, people realized that omics data may pave the way for the productivity optimization at the whole-genome scale from the perspective of systems biology [21]. In recent years, the development of high-throughput technologies has revived efforts to increase strain productivity [22-26]. For example, a new regulator of avermectin biosynthesis in *Streptomyces avermitilis*, HrdB, was identified through transcriptome profiling in our previous study, and increased productivity was achieved by modifying the *hrdB* gene [27].

The complete genome of *S. erythraea* strain NRRL23338 was sequenced in 2007 [1], which allows more rational improvement of strains to achieve high-titer erythromycin production. Several expression microarray experiments based on the wild-type strain and mutant strains including both overproducing and defected strains were later on released [6,28-30]. It was found that genes involved in erythromycin biosynthesis cluster and feeder pathway were up-regulated at the transcriptional level in overproducing strains. Even though, the global genetic basis for erythromycin biosynthesisis far from full elucidation.

In this work, we sequenced the whole genome of a high-producing strain (E3) obtained from random mutagenesis and screening, and examined time-series expression profiles of both E3 and the wild-type strain NRRL23338. A large number of genomic variations, including 60 insertions, 46 deletions and 584 single nucleotide variations (SNV) were identified in E3 in comparison with NRRL23338; the genes involved in erythromycin biosynthesis and feeder pathways were significantly up-regulated during the 60 hours time-course. According to our data, *bldD* did not show any positive correlations with the expression of *ery* cluster, implying alternative regulation mechanisms of erythromycin synthesis in *S. erythraea*, and/or the combinatorial effects of various regulation mechanisms. By integrating comparative genomic data to comparative transcriptomic data, we proposed several regulators which are potentially associated with erythromycin production. This functional comparative genomics work represents an important step towards understanding the over-producing mechanism of *S. erythraea* strain E3 on a genomic scale, and provided useful clues to strain engineering for improved production of erythromycin.

## Results

### *S. erythraea* E3 strain genome features and comparisons with NRRL23338 strain

**Figure 1:**
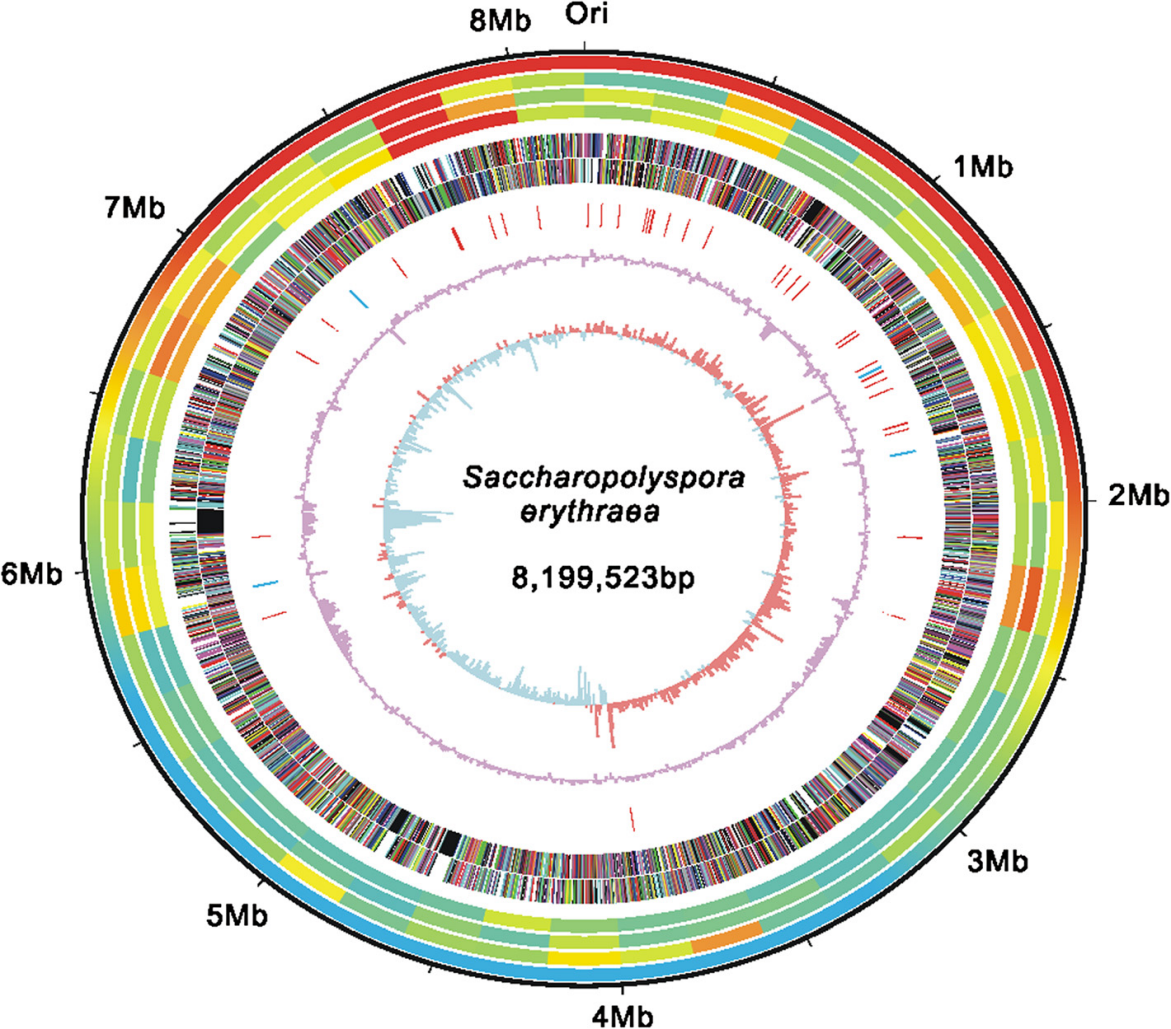
**Schematic representation of the *S. erythraea *E3 genome.** The outer scale is numbered in megabases from the origin of replication (ori) and the first circle indicates the core (red) and noncore (blue) regions, and the boundaries between them are mark by gradient colors. Circles 2, 3 and 4 (from the outside in), whole genome alignment (by Mummer) between *S. erythraea* and *M. tuberculosis, N. farcinica, S. coelicolor*, respectively (conservation of sequence regions are marked by gradient colors: blue, yellow, red. Most conserved regions are in red.). Circles 5 and 6, all genes (forward and reverse strand, respectively) colored corresponding to the COG functional assignment. Circle 7, tRNAs (red) and rRNAs (skyblue). Circle 8, GC content; circle 9, GC skew ((G – C/G + C), plum indicates values >1, lightblue values <1).

The main features of the genome sequence of high-producing strain E3 are schematically represented in Figure 1, and summarized in comparison with wild-type strain NRRL23338 in Table 1.

**Table 1:** **General features of the *S. erythraea *E3 and NRRL23338 genome**

**Component of the genome**	**NRRL23338**	**E3**
**Length**	8,212,805 bp	8,199,523 bp
**G + C content**	71.10%	71.00%
**Coding sequences**	7,264	7,257
**rRNA**	6*	12
**tRNA**	50	50
**CDSs**	7,198	7,195

CDS, protein-coding sequence. *attributed to a statistical error of the previous literature.

The genome of strain E3 is comprised of a single circular chromosome of 8,199,523 bp, which is 0.16% smaller than that of NRRL23338 (8,212,805 bp) [1]. The average GC content of the E3 genome is 71.0%, almost the same as NRRL23338 (71.1%) [1]. The replication origin of E3 genome was determined by homology mapping to NRRL23338, which was identified based on GC skew and the position of the replication origin oriC. In strain E3, there is a definite GC skew inversion at *oriC* and also on the opposite side of the chromosome to *oriC*. The initiation codon of the *dnaA* gene, adjacent to oriC, was set as the starting point for numbering the coding sequences.

The overall features of E3 genome are highly similar to NRRL23338 (Table 1). In summary, E3 contains a total of 7,257 predicted coding sequences, including 7195 protein-coding sequences (CDSs), 50 tRNA genes, and 12 rRNA genes in four copies of 16S-23S-5S rRNA operons. The coding density is 84.9%, identical to strain NRRL 23338. The 50 tRNA genes encode all tRNAs required for protein biosynthesis. The rRNA gene number of E3 strain, 12, is different from that of NRRL23338 reported earlier, 16 [1]. To address this doubt, we carefully compared the rRNA coding regions of two strains, and found that their sequences are completely identical. We then noticed that there are actually only 12 rRNA coding gene records of NRRL23338, but not the claimed 16, deposited in NCBI. All these observations suggest that there should be a total of 12 rRNA genes in both E3 and NRRL23338. Among the 7195 CDSs, a putative function could be ascribed to 4783 (66.5%) of these. Of the rest, 829 (11.5%) showed similarity to hypothetical proteins in other genomes, and 1,583 (22.0%) had no substantial similarity to predicted proteins in public databases.

Similar to the NRRL23338 strain, the core region of the genome, extending either side of the *oriC* and ending in the regions of markedly lower GC content (Figure 1), accounts for a total of 4.3 Mbp. In comparison with the regions outside the core, the core region shows substantial conservation when comparing with other actinobacteria (e.g. *M. tuberculosis, N. farcinica, S. coelicolor*, Figure 1) [1].

The genomic changes in E3 relative to NRRL23338 involve 60 insertions, 46 deletions and 584 single nucleotide variations (SNVs) (see Additional file 1: Table S1). Most of the variations occur in intragenic regions: 40 out of 60 insertions, 28 out of 46 deletions and 511 out of the 584 SNVs are located in intragenic regions, and the other 20 insertions, 18 deletions and 73 SNVs are located in intergenic regions (Additional file 1: Table S1). Based on the intragenic variations, which are expected to contribute to the altered phenotype, we identified 139 proteins whose amino acid sequence is changed by SNVs, 28 proteins and 32 proteins which are affected by deletions and insertions respectively (Additional file 2:Table S2, Additional file 3: Table S3, Additional file 4: Table S4). These altered proteins are overrepresented in the COG categories of “replication, recombination and repair” with *p*-value of 4.20E-08, and “signal transduction mechanisms” with *p*-value of 0.005.

The largest variation between E3 and NRRL23338 genomes is an 11 Kb deletion which spans 11 genes including a predicted integrase and a predicted aminoglycoside phosphotranserase as well as 9 hypothetical proteins (Additional file 3: Table S3). It is likely to be a prophage (integrated plasmid) because its gene content and organization are very similar to the isolated plasmids pSE101 and pSE211 previously identified in strain NRRL23338 [1]. Moreover, E3 genome are found to contain integrated pSE101 and pSE211, one copy for each plasmid, which include14 and 27 candidate protein-encoding genes respectively, spanning 10.9 kb and 17.3 kb [1]. It is noticeable that the integrated plasmid regions in strain E3 and the isolated pSE101 and pSE211 in strain NRRL23338 share an integrase (recombinase) gene.

### Comparative transcriptome analysis between E3 and NRRL23338 strains

Beyond the whole genome sequencing of two *S. erythraea* strains, high-producing E3 and the wild-type NRRL23338, we designed a time-course microarray experiment to investigate the gene expression profiles of E3 and NRRL23338. Six time points 10 h, 16 h, 24 h, 36 h, 48 h, 60 h were chosen according to the growth curve [28] and erythromycin production curve (Additional file 5: Figure S1) of *S. erythraea*.

**Figure 2:**
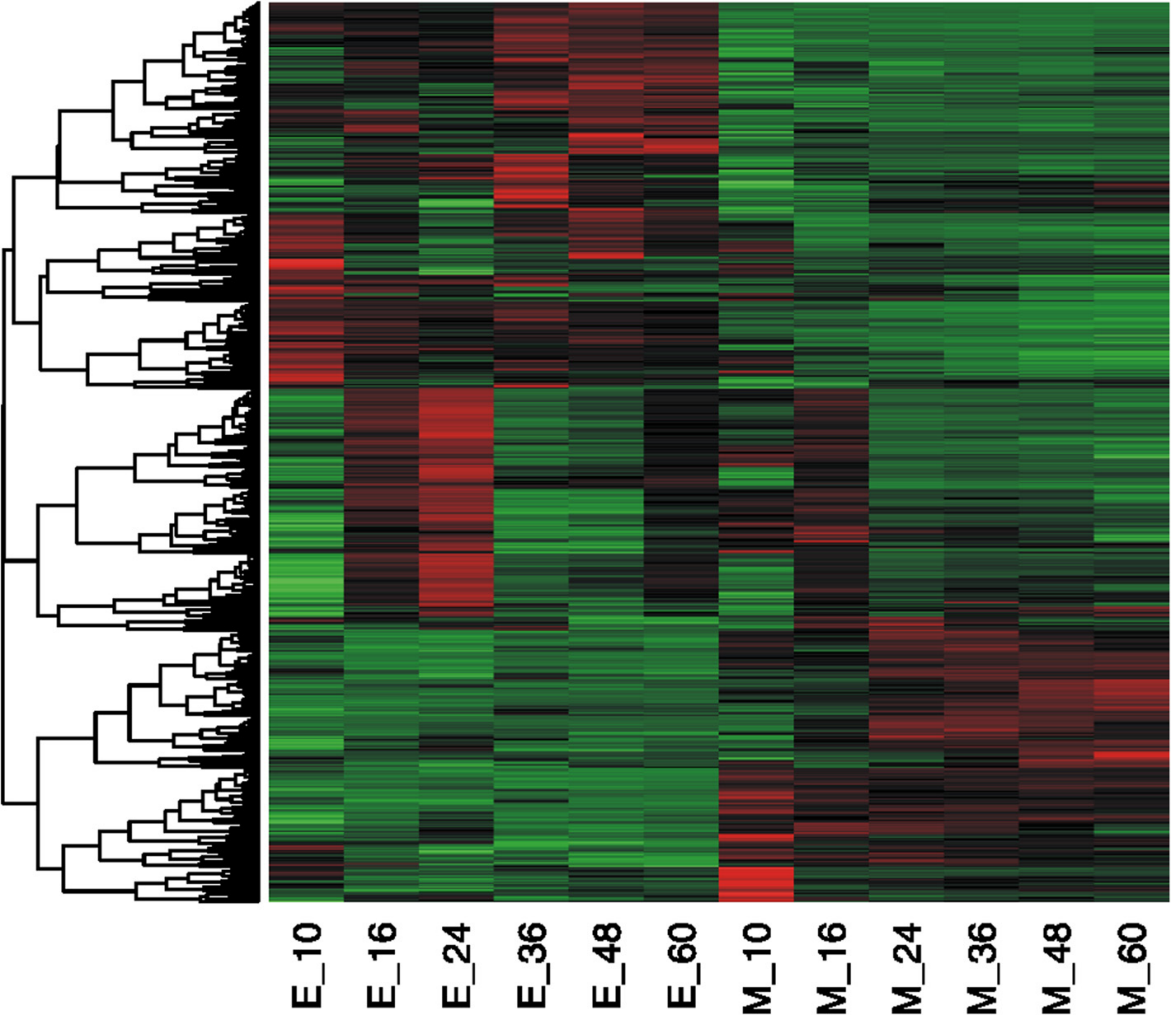
**Heatmap of the 1500 differentially expressed genes between NRRL23338 and E3.** Red = up-regulated, Green = down-regulated, the dendrogram on the left is the clustering result, the sample names (M: model strain NRRL23338, E: high-producing strain E3, the number represents the time point) are list on the bottom.

A total of 1500 genes (approximately 20% of all *S. erythraea* genes) were identified to be differentially expressed (DE) in terms of between-time series, which emphasizes on the expression variation at the same time point between two strains. The differentially expressed genes (DEGs) were then clustered into 3 groups according to the pattern of expression change: up-regulated in strain E3, down-regulated in strain E3, different expression pattern across time between two strains (Figure 2 and Additional file 6: Table S5).

According to the functional enrichment analysis results based on COG category annotation [31] (Additional file 7: Table S6), [C] energy production and conversion and [Q] Secondary metabolites biosynthesis, transport and catabolism are up-regulated in strain E3; [O] posttranslational modification, protein turnover, chaperones and [E] amino acid transport and metabolism are over-represented both in up-regulated genes and down-regulated genes in strain E3, indicating that these functions present different activities in two strains, and some of the related genes are up-regulated in E3 while some are down-regulated (Additional file 7: Table S6). Additionally, [L] replication, recombination and repair are enriched in the genes with different expression profile across time between two strains. Among them, we carefully scrutinized the specific pathways related to fermentation physiology, including erythromycin biosynthesis, feeder pathway, transporters, energy production and secondary metabolite synthesis.

### Erythromycin biosynthesis and feeder pathways

Noticeably, all genes (SACE_0712-0721, SACE_0723-0734) in erythromycin biosynthesis cluster (*ery* cluster) were dramatically up-regulated (Mean: 5.8-fold, Max: 30.8-fold) for the duration of 60 hours time-course in the overproducing strain E3 in comparison with the wild-type NRRL23338 (Additional file 8: Table S7), consistent with previous observations that the expression of *ery* cluster was correlated to erythromycin production [11,32]. Moreover, the feeder pathway was also activated in E3 (Additional file 6: Table S5, Additional file 8: Table S7). In many streptomycetes, at least four pathways have been characterized to be connected to the methylmalonyl-CoA pool (precursor of erythromycin biosynthesis): MCM pathway which catalyzes the reversible isomerization of succinyl-CoA and methymalonyl-CoA, CCR pathway utilizing crotonyl-CoA reductase or adenosylcobalamin-dependent isobutyryl-CoA mutase, MeaA pathway from acetoacetyl-CoA, and PCC pathway through carboxylation of propionyl-CoA by proprionyl-CoA carboxylase. It was found that *S. erythraea* has no CCR pathway and MeaA pathway for the precursor supply [1]. Genome sequence analysis suggests that PCC pathway may play a role in precursor flow in *S. erythraea*. In *S. erythraea* at least five genetic loci (SACE_0026-0028; SACE_3241-3242; SACE_3398-3400; SACE_3856/6509; SACE_4237) might encode biotin-dependent carboxylases catalyzing carboxylation of propionyl-CoA to methylmalonyl-CoA. So far, it is still unclear which gene set makes a contribution to erythromycin biosynthesis. Comparative transcriptome analysis found that only SACE_4237 gene displayed obvious up-regulation in high-producing strain E3, indicating that over-expression of SACE_4237 may enhance supply of methylmalonyl-CoA through carboxylation of propionyl-CoA. Further experiments are required to validate this assumption.

The expression of genes encoding key enzymes of carbon and fatty acid metabolisms was also significantly changed in turn affecting the flux of metabolites through erythromycin feeder pathways. The industrial strain E3 exhibited an impressive activation of fatty acid catabolic pathway, glycolysis/citrate cycle pathways, valine, leucine and isoleucine degradation pathway that supplies propionyl-CoA and methymalonyl-CoA for biosynthesis of 6-deoxyerythronolide B. The genes coding for key enzymes of valine, leucine and isoleucine catabolic pathway (*fadE1*, SACE_4125 coding for acyl-CoA dehydrogenase; *echA7*, SACE_4571 coding for enoyl-CoA hydratase/isomerase-like activity; *pksG*, SACE_4570 encoding hydroxymethylglutaryl-CoA synthase; *mmsA1*, SACE_4672 encoding methylmalonate-semialdehyde dehydrogenase) were strongly over-expressed in industrial strain E3. Up-regulation of genes involved in fatty acid catabolic pathway, including SACE_4038 (*fadD*) coding for long-chain acyl-CoA synthetase, SACE_4125, SACE_4571, SACE_6363 (*fadA*) encoding acetyl-CoA acetyltransferase, maybe also lead to the increased propionyl-CoA in strain E3. The glycolysis/citrate cycle pathways are connected to the feeder pathway of erythromycin biosynthesis via succinyl-CoA, an important metabolite of the Krebs cycle. Transcription of *gap*, *pgk*, *tpiA* and *eno* in glycolysis was moderately elevated in strain E3. Most genes of TCA cycle, including SACE_1638 (*sucB*), SACE_3674 (*mdh*), SACE_3811 (*acn*), SACE_3926/SACE_3927 (*korA*), SACE_3952 (*pdhA2*), SACE_3953 (*pdhB1*), SACE_3954 (*bkdC2*), SACE_4581 (*citE3*), SACE_6118 (*pyc*), SACE_6636 (isocitrate dehydrogenase) and SACE_6668/6669 (*sucC*), showed higher expression level, as compared to wild-type strain. It was found that the variations affected three genes of the Krebs cycle, including SACE_0633 (citrate synthase), SACE_6636 and SACE_6668 (Additional file 2: Table S2, Additional file 3: Table S3, and Additional file 4: Table S4).

These results demonstrate that enhanced level of erythromycin biosynthesis in strain E3 is likely to be attributed to the alterations in many pathways, and the up-regulation of erythromycin biosynthesis and feeder pathway seemed to be crucial events.

### Transporters

Differential expression analysis showed that genes encoding phosphate transport system (SACE_7097-7099, SACE_6643-6645), Iron ion uptake system (SACE_4076-4077), D-methionine transport system (SACE_0804-0806), and sulfonate/nitrate/taurine transport system (SACE_0556, SACE_1672-1674) were significantly activated, among which the transport of D-methionine (SACE_0806) was most activated (5.9 folds). PhoP-regulated *phn* operon (SACE_6643-6645) was induced by 5.5 folds. Moreover, several genes involved in nitrogen sources intake and assimilation were inhibited in E3 strain, including the oligopeptide transporter operons (SACE_0257-0260, SACE_0844-0848), amino acid transporter operon (SACE_6266-6269), glutamate transporter operon (SACE_1743-1746), glutamine transporter operon (SACE_7284-7286), and ammonium transporter gene (*amt*, SACE_6062). The most repressed gene was *amt* (12.5 folds). It was observed that the genes related to phosphate and nitrogen transport were differentially expressed between two strains. Compared with NRRL23338, E3 showed phosphate starvation response and repressive nitrogen-metabolism through the entire time-course. These observations indicated that P-N metabolism balance may play an important role in the overproducing strain E3.

### Energy production

Generally speaking, the expression of genes involved in bioenergetics and oxidative phosphorylation was moderately enhanced in industrial E3 strain, which may contribute to energy supply for erythromycin production. The most important members of this group are *nuo* operon encoding NADH dehydrogenase I (*nuoN-A,* SACE_6889-6902) and *atp* operon coding for ATP synthases (*atpDGAHFEB*, SACE_6280-6286). This is consistent with the observation that the *nuo* genes exhibited higher expression level in mutant *rif1* (higher producer) than wild type and mutant *rif6* strains (lower producer) [6].

### Secondary metabolite synthesis

**Figure 3:**
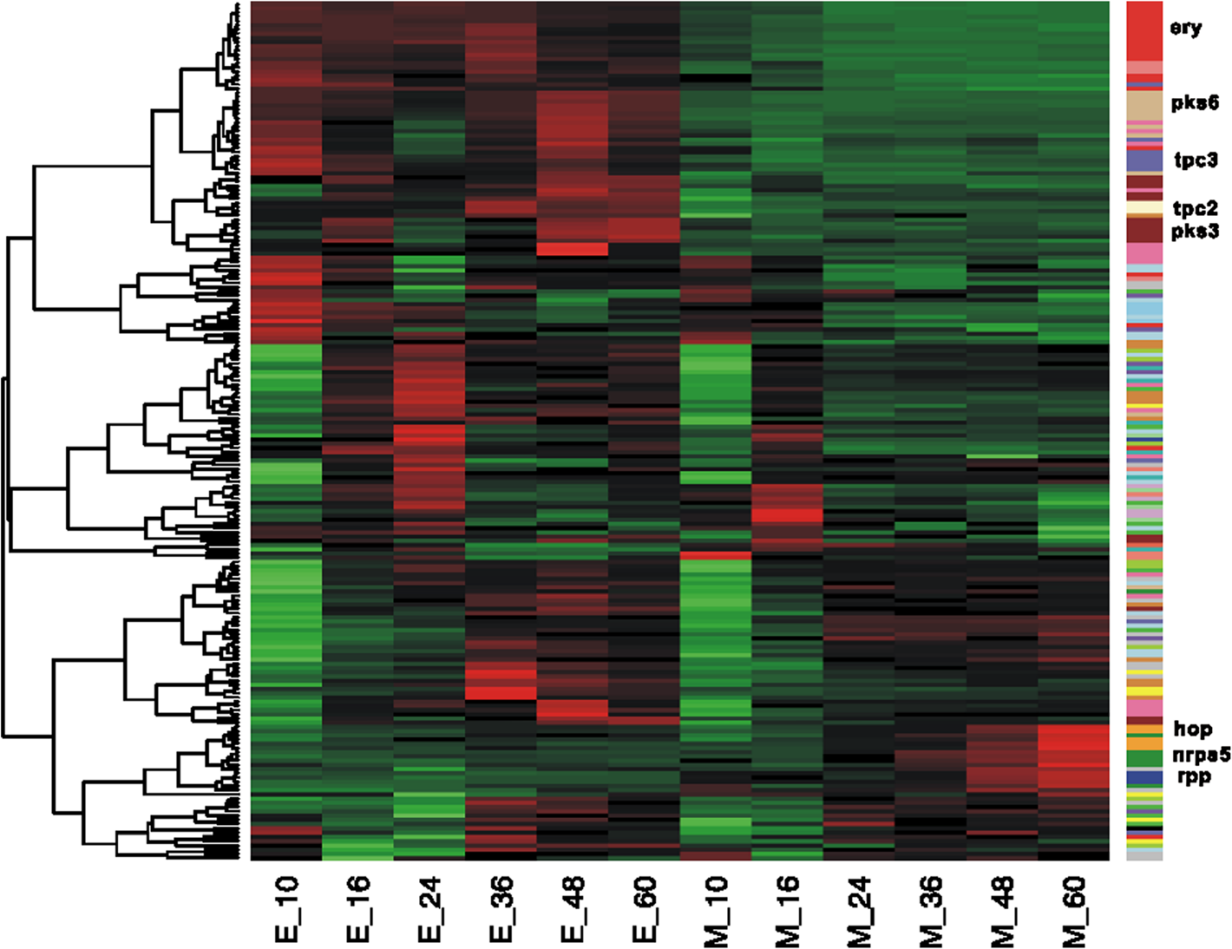
**Heatmap of secondary metabolite synthesis gene clusters between NRRL23338 and E3.** Red = up-regulated, Green = down-regulated, the dendrogram on the left is the clustering result, the sample names (M: model strain NRRL23338, E: high-producing strain E3, the number represents the time point) are list on the bottom. Genes in the same gene cluster are marked in the same color, the well clustered gene cluster *ery, tpc2, tpc3, pks3, pks6, nrps5* and *hopp* and *rpp* clusters are marked on the left.

There are 25 clusters (involving a total of 202 genes) for the biosynthesis of polyketides, terpenes and nonribosomally synthesized peptides in the *S. erythraea* genome. Three clusters (*tpc2*, *tpc3*, and *tpc4*) of the six terpene synthase gene clusters, three PKS gene clusters (*pks3*, *pke*, and *pks6*), and one gene cluster encoding nonribosomal peptide synthetase (*nrps1*) were activated in strain E3 in comparison with NRRL23338 (Figure 3). Among these up-regulated gene clusters, *pks6* (SACE_4567-4577) showed the most significantly induced expression (Mean: about 5.7 folds; Max: about 21 folds). In contrast, only two gene clusters for production of secondary metabolites (*nrps6* and *rpp*) were inhibited in E3. This indicated the general enhancement of secondary metabolite synthesis in high producing strain.

Since differential coexpression analysis (DCEA) is much more useful than traditional differential expression analysis (DEA) for investigating the global transcriptional mechanisms underlying the associated phenotypic changes [33,34], we then ranked genes according to its differential coexpression with the others in the coexpression network by using DCp method [35] (Additional file 8: Table S7). Noticeably, *Ure* operon, *nar* operon, *nir* operon, *glnA2*, *glnB*), involved in nitrogen metabolism, were differentially coexpressed, implying an altered coordination of nitrogen metabolism with the other functions in strain E3 compared with NRRL23338.

### Functional genomics analysis

After separately analyzing the genomic data and transcriptomic data, we set out to explore the links between genomic variations and transcriptomic variations, which provide an opportunity to identify causative mutations accounting for the high productivity, and eventually elucidate the regulation mechanisms underlying the erythromycin synthesis.

We first focused on the *ery* cluster genes, including the promoter regions, and found that they were identical at the genomic level in both strains, but significantly up-regulated at the transcriptional level in high-producing strain E3. We then resorted to the upstream regulatory factors of *ery* cluster. The coordinate alteration of the *ery* cluster gene expression suggested that the *ery* cluster may be co-regulated by the regulator(s), whose differences at the sequence level in two strains may lead to the expression difference of *ery* cluster. The transcriptional regulator, *bldD* (SACE_2077), which was reported to positively regulate the *ery* cluster [18], however, showed lower expression level in overproducing strain E3, opposite to the *ery* genes. This was consistent with the data reported more recently [6]. It seems that the regulatory effect of of *bldD* on *ery* cluster needs further investigation. At least, there must be other regulatory factors involved in the transcriptional regulation of *ery* genes.

We manually checked the annotation information of these genomic variant genes, and found a total of 18 potential regulators (Additional file 9: Table S8 and Additional file 10: Dataset S1). According to the comparison to the sequence of orthologous genes in closely related species, 7 genes were supposed to be mutated in E3 and 2 genes were supposed to be mutated in NRRL23338; 11 genes were uncertain as no orthologues of them were found. We focused on the two regulators, MtrA and MoxR, which were mutated in the functional domain regions in strain E3.

In *S. erythraea*, SACE_6447-SACE_6445 operon encodes proteins that are homologous to the products of the *mtrAB-lpqB* genes of *Mycobacterium tuberculosis*. MtrA is a member of the OmpR/PhoB family of response regulators, forming a two-component system (TCS) with its cognate sensor kinase (MtrB). According to NCBI conserved domain database (CDD, http://www.ncbi.nlm.nih.gov/Structure/cdd/cdd.shtml), Pfam (http://pfam.janelia.org), and the three-dimensional structure drawn from *Mycobacterium tuberculosis*[36], MtrA possesses two domains, an N-terminal regulatory domain displaying the classic α/β fold and a C-terminal DNA-binding domain with a winged helix-turn-helix. MtrA has been related to the regulation of diverse cellular functions [37-40]. In *S. coelicolor*, SCO3011-SCO3013 operon appears to be an orthologue of the *mtrAB-lpqB* operon. The function of this operon is unclear but null mutations in *mtrB* and *lpqB* affect normal septation and sporulation. Attempts to disrupt *mtrA* have so far been unsuccessful, suggesting this may also be an essential gene in *Streptomyces*[41]. Analysis of the limited experimental research available on MtrAB suggests that the primary role of MtrAB is in regulating cell cycle progression, the composition and function of the cell envelope or, perhaps more likely, in responding to general stresses that could ultimately inhibit cell division. However, many questions remain about the MtrAB two-component system in *Streptomyces*, such as: how does MtrAB govern growth and MtrAB govern growth and development? How does MtrAB play a role in antibiotic biosyntheses? To date, no experimental work has been performed on this operon, so information about its function in the cell is not available. Recently the direct repeats of GTCACAgcg-like consensus sequences (MtrA-Box) recognized by MtrA were identified in *M. tuberculosis*[42], but we failed to find it in *S. erythraea* genome, indicating that *S. erythraea* has distinct MtrA binding site upstream of the target genes [43]. Although both *mtrA and mtrB* werenot differentially expressed between two *S. erythraea* strains, MtrAofE3 strain had two amino acids deleted (H196 and V197), compared to *M. tuberculosis* and *S. erythraea* NRRL23338. The deletion was located in the C-terminal domain, and specifically, next to the DNA-binding site, V195 (Additional file 9: Table S8 and Additional file 10: Dataset S1).

We therefore applied homology modeling to study the variation sites of E3 MtrA in the structural context. It was found that the deleted sites in the *M. tuberculosis* model was in an α-helix (Additional file 11: Figure S2), which may lead to the distortion of the related helix or even more. This probably interfere its DNA binding activity, and thus the regulatory activity of MtrA. Follow-up functional experiments of MtrA in *S. erythraea* will help to identify its regulatory role in antibiotic synthesis.

We also observed another interesting regulator, MoxR (SACE_3795). The MoxR family is a subset of AAA + ATPases, a large, diverse group of ATPases associated with various cellular activities including cell-cycle regulation, DNA repair and replication, protein proteolysis and disaggregation, and so on. MoxR is often observed in close proximity to Von Willebrand Factor Type A (VWA) proteins and are likely to function with them to form a chaperone system. More importantly, MoxR also acts as DNA helicases and transcription factors [44]. In *S. erythraea*, the *moxR* gene is immediately adjacent to two genes (SACE_3794 and SACE_3793) encoding VWA proteins. A single-nucleotide variation (N133D) was identified in E3 *moxR* gene (Additional file 11: Table S2 and Additional file 12: Figure S3), compared with NRRL23338 and other related species, such as *Actinosynnema mirum* DSM 43827. When searching NCBI CDD, we found this protein was a putative member of the AAA + (ATPases Associated with a wide variety of cellular Activities) superfamily. The sequence alignment showed that the variation N133D was located in the very vicinity of ATP-binding site (two residues away) and Walker B motif (related to Mg^++^ binding, one residue away), suggesting that the substitution may perturb its activity of ATP and Mg^++^ binding, although the homology modeling result did not provide further evidence. Additionally, of the 18 regulator genes, *moxR* gene was mostly strongly over-expressed in industrial strain E3 compared with NRRL23338 (about 3-fold).

## Discussion

Erythromycin and its semi-synthetic derivatives are widely used in the clinic, and thus improved producers are highly sought after. In recent years, the availability of the entire genome sequence of *S. erythraea* has opened the possibility of defining the erythromycin biosynthesis mechanisms by using global approaches [1,28]. These high-throughput approaches have led to the discovery that BldD, a key developmental regulator in actinomycetes [16,17], which activates the synthesis of erythromycin at the transcriptional level [18]. Meanwhile, there is evidence that increasing the flux through feeder metabolic pathways strongly influences the erythromycin yields, which has been recently obtained by engineering the methylmalonyl-CoA metabolite node in *S. erythraea* and in *A. erythreum*, a non-filamentous erythromycin A producer [15,45,46].

In our present work, the comparative analysis of *S. erythraea* overproducing E3 and wild-type NRRL23338, at both genomic and transcriptomic level, indicate that *ery* clustering for erythromycin synthesis is up-regulated in E3 as expected, whereas E3 and NRRL23338 have identical *ery* genomic sequences (Figure 3). Furthermore, the feeder pathways were also activated in E3 strain. This could elevate the concentrations of propionyl-CoA and methymalonyl-CoA, the precursor metabolites of erythromycin production (starter unit and extender unit respectively), and thus improve the erythromycin yield. Indeed, increased supply of methylmalonyl-CoA has been demonstrated to increase erythromycin production, which was achieved by altering the metabolic flux distribution of its different precursors through genetic manipulation [15]. The extender unit, methymalonyl-CoA, can be derived from different pathways such as carboxylation of propionyl-CoA and rearrangement of succinyl-CoA. The *mutA* and *mutB* genes (SACE_5638, 5639) encode methylmalonyl-CoA mutase (MCM), which catalyzes the reversible isomerization of succinyl-CoA and methymalonyl-CoA. Reeves *et al.* have proposed a metabolic model where in carbohydrate-based fermentations MCM acts as a drain on the methylmalonyl-CoA metabolite pool, and in oil-based fermentations, MCM acts in the reverse direction to fill the methylmalonyl-CoA pool [45]. The induction or repression of *mutAB* may elevate or reduce concentrations of methymalonyl-CoA to affect the erythromycin synthesis rate depending on the medium. Therefore, overproduction of erythromycin was achieved by inactivating the *mutB* in a carbohydrate-based medium [15], or by duplication of the MCM operon *(mutA, mutB, meaB* and *mutR*) in an oil-based fermentation medium [20]. In this study, the carbon flow under industrial medium condition may be from succinyl-CoA to methylmalonyl-CoA. Improvement of erythromycin production in E3 strain could be at least partly attributed to the increase of methylmalonyl-CoA pool as a result of the overexpression of *mutAB* genes.

More interestingly, *bldD* has the same sequences in two strains, and is down-regulated in the overproducing E3, which is contrary to the observation reported by the BldD finder [18], while consistent with a more recent literature [6]. Other possible regulation mechanisms of erythromycin synthesis in *S. erythraea* are therefore expected.

**Figure 4:**
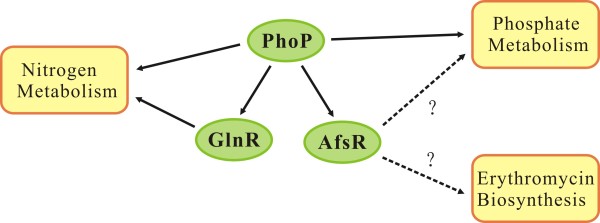
**Putative regulatory roles of PhoP, GlnR, and AfsR in phosphate, nitrogen metabolism and erythromycin biosynthesis in *S. erythraea*.**

According to our data, the differential expression between overproducer E3 and wild-type NRRL23338 significantly involved transport of phosphate and nitrogen, and phosphate starvation response and repressive nitrogen-metabolism was observed in E3 through entire time-course compared with NRRL2338 strain. This suggests that the high-level expression of erythromycin biosynthesis pathway probably result from the dysfunction of global regulators sensing stress or nutritional starvation signal, as is known in the signaling system of PhoP-AfsR-AfsS in *S. coelicolor* A3(2), controlling expression of genes involved in P-N nutrition stress response and secondary metabolism [47]. AfsS, a 63-amino-acid sigma-like regulatory protein, was found in streptomycetes including *S. coelicolor, S. dividans, S. griseus, and S. noursei*. In *S. coelicolor,* the activation of AfsS by AfsR can further activate the transcription of genes coding for pathway-specific transcription factors, *e.g. actII-ORF4* and Moreover, AfsS, as a crucial master regulator of both antibiotic synthesis and nutritional stress response, also regulates the expression of genes involved in phosphate transport and nitrogen metabolism. The global network of signal transduction cascades and cross-talk of PhoP and AfsR-AfsS was modeled recently, which controls gene expression involved in P-N balance and secondary metabolism in *S. coelicolor*[48-50]. PhoP repressed the transcription of nitrogen genes by binding to the promoter of *glnR*, the major nitrogen regulator, to the promoters of *glnA* and *glnII*, two glutamine synthetases, and to the promoter of the *amtB-glnK-glnD* operon, encoding an ammonium transporter [51]. It is interesting that the *S. erythraea* orthologs of these genes and other GlnR-regulon genes were significantly down-regulated in strain E3, such as *glnR* (SACE_7101), *glnB* (SACE_6061) encoding the nitrogen regulatory protein PII, *amt* (SACE_6062), *glnA-1* (SACE 1623) coding for the glutamine synthetase, *ure* operons (SACE_0634-0636, SACE_2526-2527) coding for the urease, *narK-nir* operon (SACE_3799-3803) encoding the assimilatory nitrite reductase and nitrite extrusion protein. The previous works also demonstrated that many genes related to nitrogen metabolism showed lower expression in *S. erythraea* overproducing strain *rif1* with S444F mutation in *rpoB* than in impaired strain *rif6* with Q426R mutation [6]. In order to investigate if these nitrogen metabolism genes are under the control of PhoP in *S. erythraea*, we searched these genes for PHO boxes using the model based on the alignment of 25 PHO DRu compiled by Blanco *et al*. [52], and detected one candidate PHO box (two DRus: GTTCGCCTTCTGTTCACAATTG) in the *glnR* promoter region, and one (two DRus: CTTCCCGTGCCGTTCAGCAACG) in the *afsR* promoter. However, we failed to find sequences coding AfsS-like protein in *S. erythraea* genome. So far the signaling system of PhoP-AfsR-AfsS, controlling expression of genes involved in P-N nutrition stress response and secondary metabolism in *S. erythraea* has not been built. Our findings suggest the existence of a similar regulation mechanism underlying the erythromycin biosynthesis in *S. erythraea* (Figure 4).

Taken together, it seems that the regulatory mechanism of erythromycin biosynthesis is much more complicated than expected, probably involving more than one global regulator. Further function experiments, such as the identification of the regulators and their target genes, the identification of PhoP-AfsR-AfsS signaling system, will help to elucidate the precise mechanisms. Furthermore, the accumulation of genomic and transcriptomic data for more over-producing strains will also help to find out the causal variations which directly contribute to antibiotic production. Actually, a comparative genomics and transciptomic profiles of another erythromycin over-producing strain Px and NRRL2338 was released very recently [53]. It was found that the genomic variations affect a total of 227 proteins of Px strain and a quite number of mutations alter key enzymes in the central carbon and nitrogen metabolism and in the biosynthesis of secondary metabolites, which probably results in the redirection of common precursors for erythromycin biosynthesis [53]. Similar to the current report, it is difficult to sort out the mutations which are directly related to the high production of erythromycin. Besides experimental verification, next efforts on the comparison of different overproducers by using advanced data integration techniques will help to generate more insights on the regulation mechanism of erythromycin biosynthesis, and eventually provide valuable information on new strategies for strain improvement.

## Conclusions

In this work, we sequenced the whole genome of a high-producing *S. erythraea* strain E3 obtained by random mutagenesis and screening from the wild-type strain NRRL23338, and examined time-series expression profiles of both E3 and NRRL23338. Based on the genomic data and transcriptpmic data of these two strains, we carried out comparative analysis of high-producing strain and wild-type strain at both the genomic level and the transcriptomic level. A large number of genomic variations, including 60 insertions, 46 deletions and 584 single nucleotide variations (SNV) were identified in E3 in comparison with NRRL23338, and the analysis of time series transcriptomic data indicated that the genes involved in erythromycin biosynthesis and feeder pathways were significantly up-regulated during the 60 hours time-course. According to our data, BldD, a previously identified *ery* cluster regulator, did not show any positive correlations with the expression of *ery* cluster, suggesting the existence of alternative regulation mechanisms of erythromycin synthesis in *S. erythraea*. Several potential regulators were then proposed by integration analysis of genomic and transcriptomic data. This functional comparative genomics work represents an important step towards understanding the over-producing mechanism of *S. erythraea* strain E3 on a genomic scale, and provided useful clues to strain engineering for improved production of erythromycin.

## Methods

### Bacterial strains and culture conditions

Two *S. erythraea* strains, the wild-type NRRL23338 and a high-producing strain E3, were used in this study. *S. erythraea* strains were grown on agar plates of the medium [10 g cornstarch, 10 g corn steep liquor, 3 g NaCl, 3 g (NH_4_)_2_SO_4_, 2 g CaCO_3_, and 2 g agar per liter of distilled H_2_O, pH 7.2] at 34°C for sporulation. An agar piece about 1 cm^2^ was inoculated into a 500 ml flask containing 50 ml of the seed medium [50 g/L cornstarch, 18 g/L soybean flour, 13 g/L corn steep liquor, 3 g/L NaCl, 1 g/L (NH_4_)_2_SO_4_, 1 g/L NH_4_NO_3_, 5 g/L soybean oil, and 6 g/L CaCO_3_, pH 6.8 to 7.0] and grown for 48 h at 34°C and 200 rpm, then added 5 ml of the seed culture to a 500 ml flask containing 60 ml of the fresh industrail fermentation medium, incubation was continued at 34°C and 200 rpm for 4 days. The industrail medium consists of 40 g/L cornstarch, 30 g/L soybean flour, 30 g/L dextrin, 2 g/L (NH4)_2_SO_4_, 10 g/L soybean oil, and 6 g/L CaCO_3_ (pH 7.0 to 7.2). All media types were sterilized by autoclaving at 121°C for 30 min. Both supernatant and cell pellet samples (1 ml) were harvested at six different time points (10 h, 16 h, 24 h, 36 h, 48 h, and 60 h) during 4 days culture period, and erythromycin titers and biomass were determined. Mycelium for RNA isolation were immediately quenched in liquid nitrogen, and stored at −80°C until further use. Two independently cultured replicates were performed for the two strains fermentation experiment.

### Erythromycin titer measurements

The amount of the erythromycin produced in the fermentation broth was determined by means of biological method. Bioassays for erythromycin were performed using a large plate double-agar layer system. The bottom agar layer consisted of the test medium (5 g peptone, 3 g beef extract, 3 g K_2_HPO_4_, and 15 g agar per liter of distilled H_2_O) without *Bacillus pumilus* culture. Once the bottom layer was solidified, a top agar layer (contains *B. pumilus* culture) was poured. After the upper layer was solidified at room temperature, fermentation supernatant (260 μl) was added to stainless steel cylinders on agar plates. The bioassay plates were incubated overnight at 37°C. After incubation, the erythromycin production was estimated by measuring the diameters of the inhibition zones and calculated according to the calibration curve made by using the commercially available erythromycin as a control. Whereas two strains grew at similar rates, the E3 strain produced about 30-fold more erythromycin (600 mg/L) than the NRRL23338 strain (20 mg/L) (Additional file 5: Figure S1).

### Genome sequencing and assembly

The nucleotide sequence of the *S. erytheraea* strain E3 genome was determined by using a massively parallel pyrosequencing technology (Roche 454 GS FLX). A total of 273 contigs (>500 bp) with a total size of 8.2 Mb were assembled from 876,307 reads (average length of 238 bp) using Newbler software of the 454 suite package, providing a 25-fold coverage. Relationship among contigs was determined using the genome of *S. erytheraea* NRRL 23338 as reference, and validated by PCR. Gaps between contigs were filled by sequencing PCR products. The final sequence assembly was carried out using phred/phrap/consed package (http://www.phrap.org/phredphrapconsed.html), and all the low sequence quality regions, including homopolymeric sites, were resequenced using PCR Sanger sequencing. The final sequencing accuracy of genome was 99.9965%.

### Genome annotation and analysis

CDSs were predicted by using Genemark (http://exon.biology.gatech.edu/) [54] and Glimmer (http://ccb.jhu.edu/software/glimmer/index.shtml) [55], and manually curated. Functional annotation of CDSs was performed through comparisons to NCBI nr database using BLASTP (2.2.14) [56] and classified according to COGs (http://www.ncbi.nlm.nih.gov/COG/) [31] and Gene Ontology (http://www.geneontology.org/) [57]. Transfer RNA genes were predicted with tRNAscan-SE [58]. Whole genome alignment between strain E3 and NRRL23338 were performed by using BLASTN (2.2.14) [56].

### Microarray construction and transcript profiling

The *S. erythraea* DNA microarrays (SER v1.0) were customized using Agilent eArray 6.0 according to the manufacturer’s recommendations (https://earray.chem.agilent.com/earray/).

Each customized microarray (8x15K) contained spots in duplicate with 7,198 gene-specific 60-mer oligonucleotide probes interrogating the 7,198 predicted ORFs in *S. erythraea* (as reported for the *S. erythraea* genome at http://131.111.43.95/gnmweb/index.html). RNA was extracted from mycelium pellets deriving from two independent culture samples using the Column Plant RNAout (TIANDZ) according to the standard protocols. The RNAs were subsequently purified by QIAGEN RNeasy Mini Kit. The quality and quantity were determined by nanodrop UV spectroscopy (Ocean Optics) and analysis on a RNA 6000 Nano LabChip (Agilent Technologies) using a 2100 bioanalyzer (Agilent Technologies). RNA samples were processed and hybridized to the customized chips SER v1.0.

### Data processing and analysis

Data quality assessment and normalization were performed by using limma package in Bioconductor [59]. The differentially expressed genes were identified with timecourse package [60] in terms of between- time series by comparing time-course mean profiles with multivariate empirical Bayes model [61].

A recently published differential coexpression analysis method (‘DCp’) was applied to explore differentially coexpressed genes (DCGs) [35]. When constructing the coexpression networks for two contrastive strains, the correlation cutoff was set as 0.8 to filter coexpression links. The differentially coexpressed genes (DCGs) were ranked according to their p values.

### Protein structure modeling

Homology modeling was applied to predict three-dimensional structures of the interested proteins. We first searched PDB (http://www.rcsb.org) for putative structural templates using NCBI BLAST [62] with sequence identity greater than 30%. After that, only the best template was kept according to the alignment coverage, sequence identity and structural resolution in combination. MODELLER was then adopted to concisely align the interested protein to its template, and build the three-dimensional homology model automatically [63]. DOPE score, as suggested by the manual of MODELLER, was used to sort out the best structure model from all those generated. The selected models were analyzed in PyMOL (http://www.pymol.org/).

### Motif search method

The positional weight matrix (PWM) was generated according to the literature [64]. Upstream 300 bp and downstream 50 bp from the gene start positions were scanned by Patser to find potential target regulated genes and TFBSs [65].

## Competing interests

The authors declare that they have no competing interests.

## Authors’ contributions

YWB, LH and ZY carried out the molecular genetics experiments. CX and LYY performed the data analysis. YZQ, YH and LBH also contribute to the data analysis. LYX, YBC and LYY conceived of the study, and participated in its design and coordination. LYY, YBC and CX draft the manuscript. ZSL contributed to the coordination. All authors read and approved the final manuscript.

## Supplementary Material

Additional file 1: Table S1 All nucleotide variations detected in E3 compared with NRRL23338. Click here for file

Additional file 2: Table S2 Single amino acid substitution detected in E3 compared with NRL23338. Click here for file

Additional file 3: Table S3 Amino acid deletion detected in E3 compared with NRRL23338. Click here for file

Additional file 4: Table S4 Amino acid insertion detected in E3 compared with NRRL23338. Click here for file

Additional file 5: Figure S1 Erythromycin production curve during the time-course for the industrial *S. erythraea* strain and the wild-type NRRL23338 strain. E1 and E2 are two replicates for the industrial strain; M1 and M2 are two replicates for the wild-type strain. Click here for file

Additional file 6: Table S5 Genome annotation of *S. erythraea* E3 strain. Click here for file

Additional file 7: Table S6 COG classification of differentially expressed genes. Click here for file

Additional file 8: Table S7 Gene expression data. Click here for file

Additional file 9: Table S8 Regulators involving amino acid variation. Click here for file

Additional file 10: Dataset S1 Sequence alignment results of 18 regulators. Click here for file

Additional file 11: Figure S2 Structural Models of MtrA (V197 H198 deletion): SACE_6447 and ETHR_6255. His197 and Val198 of SACE_6447 **(A)**, deleted in ETHR_6255 **(B)**, are located in the center of an α-helix. The deletions are supposed to break the helix and to perturb the DNA-binding function of Val196 in the active state. In addition, His197 contributes to the inter-domain interactions in the inactive state, and its deletion would thus affect the stability of ETHR_6255. Click here for file

Additional file 12: Figure S3 Structural Models of MoxR (N133D): SACE_3795 and ETHR_3703. The Asn133 in SACE_3795 **(A)**, substituted by Asp133 in ETHR_6255 **(B)**, is located in the vicinity of Asp130 and Asn172, both of which were annotated as ATP-binding sites according to NCBI CDD. The Asn133 may thereby be implicated in the ATP-binding through the proxy of Asp130 and Asn172, or even through direct interaction with ATP. In addition, the nearby residues 128–131 are also putative ‘Walker B motif’, which is important for interacting with Mg^2+^ cation. So the substitution of Asn133 to Asp133, resulting in negative charge on the side chain, may affect the ATP-binding or Mg^2+^ cation-binding of the protein and thus its catalytic capability. Click here for file
