# Supplementary figures and images for: Systems perspectives on erythromycin biosynthesis by comparative genomic and transcriptomic analyses of S. erythraea E3 and NRRL23338 strains
Source: BMC Genomics. 2013 Jul 31;14:523. doi: 10.1186/1471-2164-14-523 (PMC3733707; doi:10.1186/1471-2164-14-523)

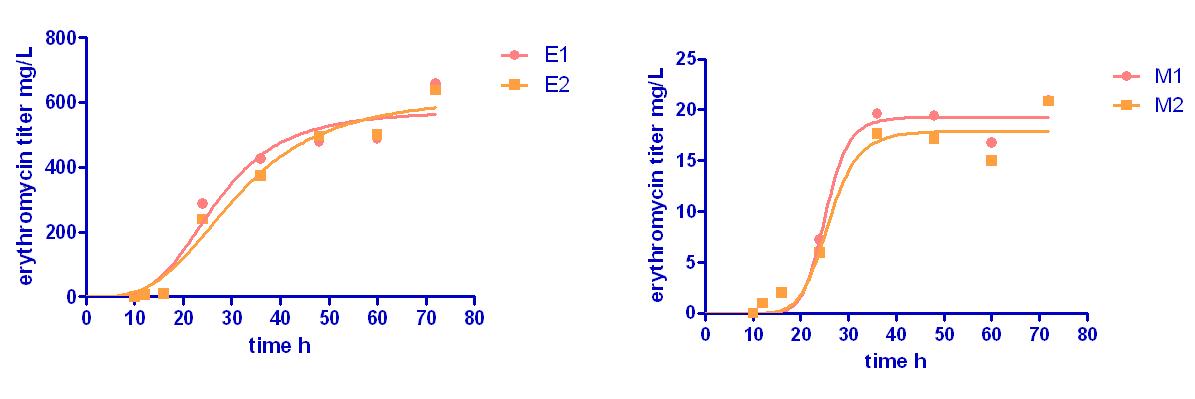

Supplement: Additional file 5: Figure S1 — Erythromycin production curve during the time-course for the industrial S. erythraea strain and the wild-type NRRL23338 strain. E1 and E2 are two replicates for the industrial strain; M1 and M2 are two replicates for the wild-type strain. [file 1471-2164-14-523-S5.jpeg]

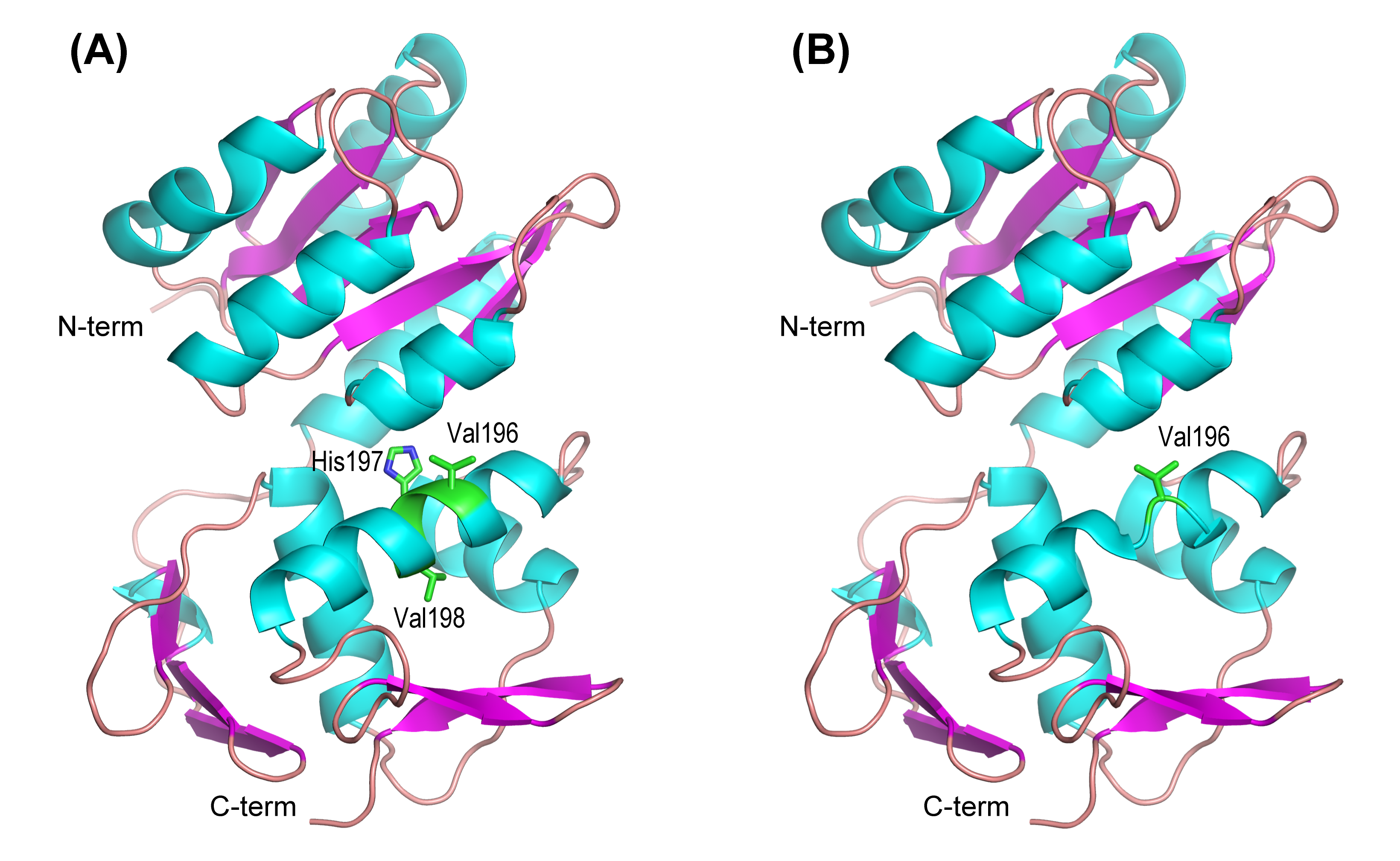

Supplement: Additional file 11: Figure S2 — Structural Models of MtrA (V197 H198 deletion): SACE_6447 and ETHR_6255. His197 and Val198 of SACE_6447 (A), deleted in ETHR_6255 (B), are located in the center of an α-helix. The deletions are supposed to break the helix and to perturb the DNA-binding function of Val196 in the active state. In addition, His197 contributes to the inter-domain interactions in the inactive state, and its deletion would thus affect the stability of ETHR_6255. [file 1471-2164-14-523-S11.tiff]

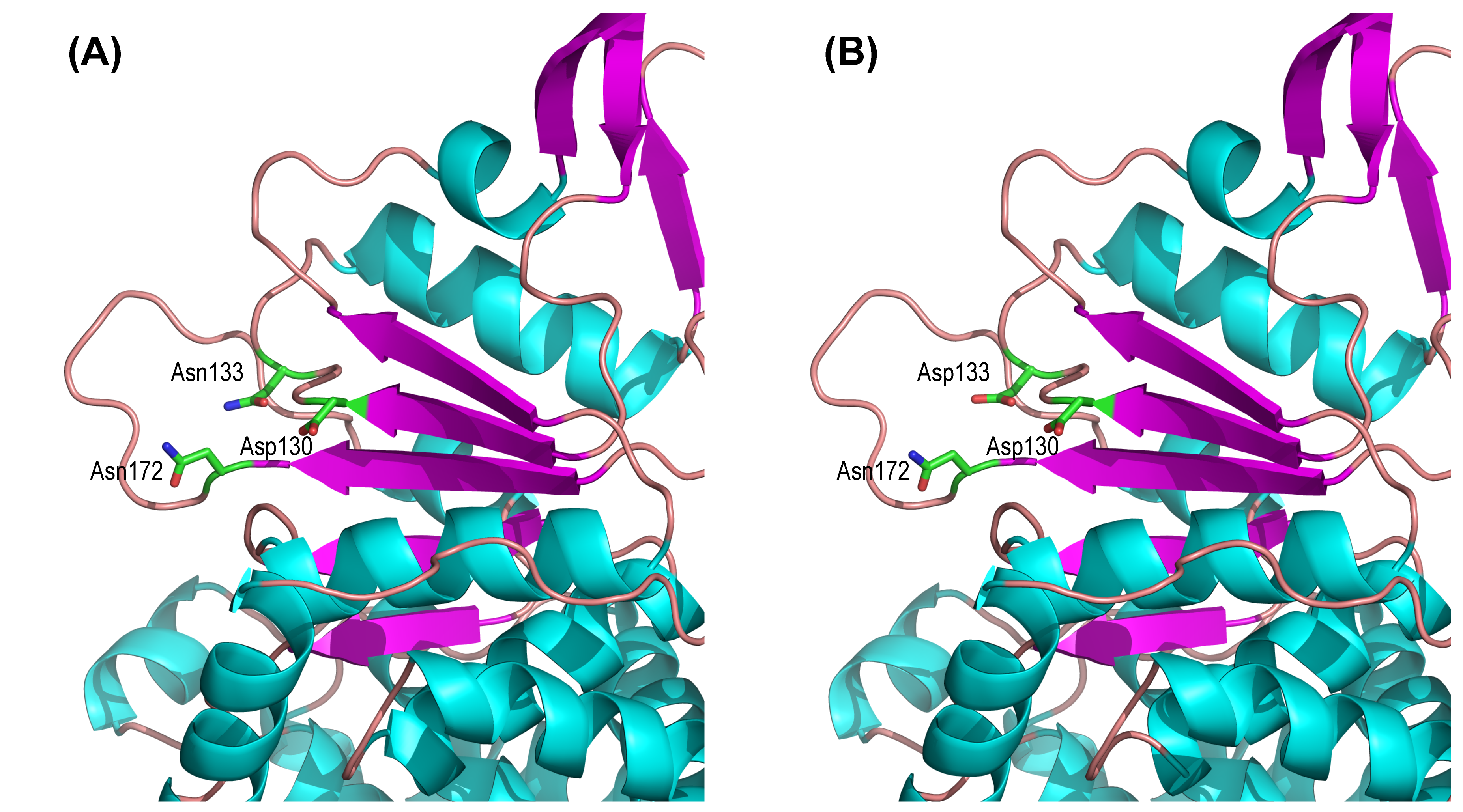

Supplement: Additional file 12: Figure S3 — Structural Models of MoxR (N133D): SACE_3795 and ETHR_3703. The Asn133 in SACE_3795 (A), substituted by Asp133 in ETHR_6255 (B), is located in the vicinity of Asp130 and Asn172, both of which were annotated as ATP-binding sites according to NCBI CDD. The Asn133 may thereby be implicated in the ATP-binding through the proxy of Asp130 and Asn172, or even through direct interaction with ATP. In addition, the nearby residues 128–131 are also putative ‘Walker B motif’, which is important for interacting with Mg2+ cation. So the substitution of Asn133 to Asp133, resulting in negative charge on the side chain, may affect the ATP-binding or Mg2+ cation-binding of the protein and thus its catalytic capability. [file 1471-2164-14-523-S12.tiff]
