# Supplementary material for: Systems perspectives on erythromycin biosynthesis by comparative genomic and transcriptomic analyses of S. erythraea E3 and NRRL23338 strains
Source: BMC Genomics. 2013 Jul 31;14:523. doi: 10.1186/1471-2164-14-523 (PMC3733707; doi:10.1186/1471-2164-14-523)
Supplement: Additional file 10: Dataset S1 — Sequence alignment results of 18 regulators. [file 1471-2164-14-523-S10.doc]

Sequence alignment results of 18 regulators:

1、Ref: No; change: uncertain; domain: No

SeqA Name Len(aa) SeqB Name Len(aa) Score

=========================================================

1 ETHR_0858 261 2 SACE_0891 206 94

=========================================================

ETHR_0858 MARKRLTREESRQETRARLLESAAELFAERGVNGASVEQIAERAGYSRGAFYGNFADKHE 60

SACE_0891 MARKRLTREESRQETRARLLESAAELFAERGVNGASVEQIAERAGYSRGAFYGNFADKHE 60

************************************************************

ETHR_0858 LVVELLRQRTLRELDEVTAIAREPEPFAALREWHKERAEHLVGWLSLRAELLLYALRTPS 120

SACE_0891 LVVELLRQRTLRELDEVTAIAREPEPFAALREWHKERAEHLVGWLSLRAELLLYALRTPS 120

************************************************************

ETHR_0858 FRPQMAERERIARDAHAGSIEATFASLGVEPPADPAFLALIVHALEDGLLVQRLLSPDEV 180

SACE_0891 FRPQMAERERIARDAHAGSIEATFASLGVEPPADPAFLALIVHALEDGLLVQRLLSPDEV 180

************************************************************

ETHR_0858 SDEVVVDAVELLMRLVVGGRGGEGRHLSPSTRPGVRSGRRGHEEITSGPPRWPAPEIGSG 240

SACE_0891 SDEVVVDAVELLMR---------------------------------------------- 194

**************

ETHR_0858 AWHPGRADPAGTAVQVRPGMT 261

SACE_0891 SWSAAGAAKGDT--------- 206

:* .. * ..*

2、Ref: 1; Change: NRRL23338; domain: No

SeqA Name Len(aa) SeqB Name Len(aa) Score

=========================================================

1 ETHR_1781 238 2 SACE_1833 241 93

1 ETHR_1781 238 3 Amir_1549 238 95

2 SACE_1833 241 3 Amir_1549 238 88

=========================================================

ETHR_1781 MAAVGLGQAARTTPAGALPANMVPHPREELFTVLVVDDHPLLREAIAARLLQMGAGTVHE 60

Amir_1549 MAAVGLGQAVRTTPAGSLPANMVPHPREELFSVLVVDDHPLLREAISARLTQMGAGTVHE 60

SACE_1833 MAAVGLGQAARTTPAGALPANMVPHPREELFTVLVVDDHPLLREAIAARLLQMGAGTVHE 60

*********.******:**************:**************:*** *********

ETHR_1781 AASMAEARARALATGPCDLAILDLGLPDGTGIDLVTELRTQGWPRIVVLASSDDPYAVRA 120

Amir_1549 AASVAEARARALATGPCDLAILDLGLPDGTGIDLVTELRAQGWPRIVVLASSDDPYAVRS 120

SACE_1833 AASMAEARARALATGPCDLAILDLGLPDGTGIDLVTELRTQGWPRIVVLASSDDPYAVRA 120

***:***********************************:*******************:

ETHR_1781 AFQAGAQAYLLKSASPMVVTDGVRRVLDGGVYADPSVAPVLAAGTRVPGTDNTPRELSGR 180

Amir_1549 AFQAGAQAYLLKSASPMVVTDGVRRVLDGGVYADPSVAPVLAAGTRVPGTDNTPRELSAR 180

SACE_1833 AFQAGAQAYLLKSASPMVVTDGVRRVLDGGVYADPSVAPVLAAGTRVPGTDNTPRELSGR 180

**********************************************************.*

ETHR_1781 EVEVLQLVADGRSNKEIGEALNLSALTVKSHLSRIGRKLGTG--DRAQMVALAMRAGVIR 238

Amir_1549 EVEVLQLVADGQSNKEIGEALSLSALTVKSHLSRIGRKLGTG--DRAQMVALAMRAGVIR 238

SACE_1833 EVEVLQLVADGRSNKEIGEALNLSALTVKSHLSRIGRKLGTGRPGSDGRPGHARRGDQMT 240

***********:*********.******************** . . * *.. :

ETHR_1781 -

Amir_1549 -

SACE_1833 G 241

3、Ref: 1; change: E3; domain: Yes

SeqA Name Len(aa) SeqB Name Len(aa) Score

=========================================================

1 ETHR_6255 226 2 SACE_6447 228 100

1 ETHR_6255 226 3 Amir_6310 261 92

2 SACE_6447 228 3 Amir_6310 261 92

=========================================================

ETHR_6255 ---------------------------------MWGMKARVLVVDDDPALAEMLTIVLRG 27

SACE_6447 ---------------------------------MWGMKARVLVVDDDPALAEMLTIVLRG 27

Amir_6310 MDRRRALVTDRDRVTTSGDERWAVREKRAGSGTMFTMKARVLVVDDDPALAEMLTIVLRG 60

*: ************************

ETHR_6255 EGFETAVVSDGTKAMPALRELKPDLVLLDLMLPGMNGIDVCKAIRTESVVPIVMLTAKSD 87

SACE_6447 EGFETAVVSDGTKAMPALRELKPDLVLLDLMLPGMNGIDVCKAIRTESVVPIVMLTAKSD 87

Amir_6310 EGFDTAVVADGARALPALRELKPDLVLLDLMLPGMNGIDVCKAIRSESGVPIVMLTAKSD 120

***:****:**::*:******************************:** ***********

ETHR_6255 TVDVVLGLESGADDYVVKPFKPKELVARLRARLRRTDAEPAEVLSIGDLTIDVPGHEVTR 147

SACE_6447 TVDVVLGLESGADDYVVKPFKPKELVARLRARLRRTDAEPAEVLSIGDLTIDVPGHEVTR 147

Amir_6310 TVDVVLGLESGADDYVVKPFKPKELVARIRARMRRTEAEPAEVLQIGDLTIDVPGHEVLR 180

****************************:***:***:*******.************* *

ETHR_6255 DGQPIALTPLEFDLLVALARKPRQVFTREVLLEQVWGYRHAADTRLVN--VQRLRSKVER 205

SACE_6447 DGQPIALTPLEFDLLVALARKPRQVFTREVLLEQVWGYRHAADTRLVNVHVQRLRSKVER 207

Amir_6310 EGRPIQLTPLEFDLLVALARKPRQVFTREVLLEQVWGYRHAADTRLVNVHVQRLRSKVER 240

:*:** ****************************************** **********

ETHR_6255 DPERPEVVLTVRGVGYKAGPP 226

SACE_6447 DPERPEVVLTVRGVGYKAGPP 228

Amir_6310 DPEHPEVVLTVRGVGYKAGPP 261

***:*****************

4、Ref: No; change: uncertain; domain: No

SeqA Name Len(aa) SeqB Name Len(aa) Score

=========================================================

1 ETHR_1841 304 2 SACE_1895 281 73

=========================================================

ETHR_1841 MSGPTIRRRQLGRQLRQLRDAAGKSSRAAAEWLDVSQATVSKVENGKQAPKIAHVRLLLQ 60

SACE_1895 MSGPTIRRRQLGRQLRQLRDAAGKSSRAAAEWLDVSQATVSKVENGKQAPKIAHVRLLLQ 60

************************************************************

ETHR_1841 LYGVESPRSEAILRLAREANQRGWWTAYGDTVPDWFRDYVGLETDATELWTYDAELIHGL 120

SACE_1895 LYGVESPRSEAILRLAREANQRGWWTAYGDTVPDWFRDYVGLETDATELWTYDAELIHGL 120

************************************************************

ETHR_1841 LQTPDYIRAVTAACNEESADFDVECYIDQRQARQDRLTSSNPPLFSAVVSEGALRRMVGG 180

SACE_1895 LQTPDYIRAVTAACNEESADFDVECYIDQRQARQDRLTSSNPPLFSAVVSEGALRRMVGG 180

************************************************************

ETHR_1841 PEVMRAQIQHLIELSKLPHVTLPGAAVRGGRAPGDGLAVHPARFRGGPGHGCGLPGEPPG 240

SACE_1895 PEVMRAQIQHLIELSKLPHVTFQVLPFEAGAHPAMVSPFILLGFEEDPDMGAVYLENHQG 240

*********************: ....* *. .. *. .*. *. : *

ETHR_1841 RALPGASGRCPALRQHLRAGPESGFDAEENEGLSRYPGAVHVTPRERSAGGERLIPRRMA 300

SACE_1895 -------------ALYLERPDDVRLYANTFERVR----KAALTPKKT---KDFLVT--LG 278

:*. : : *: * : . :**:: : *:. :.

ETHR_1841 QVLP 304

SACE_1895 QSM- 281

* :

5、Ref: No; change: uncertain; domain: No

SeqA Name Len(aa) SeqB Name Len(aa) Score

=========================================================

1 ETHR_4283 279 2 SACE_4376 900 99

=========================================================

ETHR_4283 ------------------------------------------------------------

SACE_4376 MTGPAEPLIGQGDALAELEGLVREAGEHRCGFVVVSGAAGTGKSTLGRRLLARHEGPAWW 60

ETHR_4283 ------------------------------------------------------------

SACE_4376 ATAAPWESRQPYGVVAQMLPDLDVGAGPVAVAERVADRLAASDGTTVAVVDDGHWADEES 120

ETHR_4283 ------------------------------------------------------------

SACE_4376 LQALASVVRHHPRARLLVVAFAVTGDPHASAATLELLPRIATAEIRLSPLTAAQVNELAA 180

ETHR_4283 ------------------------------------------------------------

SACE_4376 ARGVVLHPSIAERLCHHTQGAARHIAQLLDEVPAATWTRFDPGLPAPAAVAARVRELLAG 240

ETHR_4283 ------------------------------------------------------------

SACE_4376 CSPTVRAFVEATAVLGPGTAVRDAALLAGVDDGLLTVLDEACATGLVRLAPRGLAEVGPA 300

ETHR_4283 ------------------------------------------------------------

SACE_4376 DPMVRAAVLAALAPGRRSGLHRRAADLVDDPARKLRLLVAASPVPDPAVADRLDALATER 360

ETHR_4283 ------------------------------------------------------------

SACE_4376 ASEGAWAEAASLLIDASRLTEDRLERESRISRAVDALIGAGDVSAAAALVPEVESLRETP 420

ETHR_4283 ------------------------------------------------------------

SACE_4376 LRNAVLGYLAVVRGRVAEAESRLGRAWSLVNAERDPDVAALICQRYVLHALVRCRAGELV 480

ETHR_4283 ------------------------------------------------------------

SACE_4376 TWADRAISLAGPDAPAAVEAAAIRGLGLAATGRAGQARRDYARLGERVRHGAQAQRVMMA 540

ETHR_4283 ------------------------------------------------------------

SACE_4376 RGWLDLLTDAVDDARAELESAVPTTFLGGSTRISLWARAWLARAQLLAGAWDDAVRTVRD 600

ETHR_4283 ---------------------MTVHALRGEWEQAEQALLRCDAGPQDYEIMRVPTYLARA 39

SACE_4376 AAPLLERSGIVLTGPLLHWTEVTVHALRGEWEQAEQALLRCDAGPQDYEIMRVPTYLARA 660

:**************************************

ETHR_4283 QVAEARADSAGVLRALHPLTQSWAGGGIDEPGHWPWHDMYAHALVLEGRHEEADEFLLPH 99

SACE_4376 QVAEARADSAGVLRALHPLTQSWAGGGIDEPGHWPWHDMYAHALVLEGRHEEADEFLLPH 720

************************************************************

ETHR_4283 ERLAATREHASARARLAAARGRLLGAQGDPAAATTAFEQALELLEGLPLRYDHARVAFAY 159

SACE_4376 ERLAATREHASARARLAAARGRLLGAQGDPAAATTAFEQALELLEGLPLRYDHARVAFAY 780

************************************************************

ETHR_4283 GQTLRRAGKRREADAVLTTARDGFAAMGAATYVERCDRELKAGGVHTLRAERAFNELTPQ 219

SACE_4376 GQTLRRAGKRREADAVLTTARDGFAAMGAATYVERCDRELKAGGVHTLRAERAFNELTPQ 840

************************************************************

ETHR_4283 EEAVARLVARGLSNREAAAELFLSTKTVQYHLTRIYTKLGIRSRAELAALRSASEDPNRE 279

SACE_4376 EEAVARLVARGLSNREAAAELFLSTKTVQYHLTRIYTKLGIRSRAELAALRSASEDPNRE 900

************************************************************

6、Ref: No; change: uncertain; domain: No

SeqA Name Len(aa) SeqB Name Len(aa) Score

=========================================================

1 ETHR_4284 629 2 SACE_4376 900 87

=========================================================

ETHR_4284 MTGPAEPLIGQGDALAELEGLVREAGEHRCGFVVVSGAAGTGKSTLGRRLLARHEGPAWW 60

SACE_4376 MTGPAEPLIGQGDALAELEGLVREAGEHRCGFVVVSGAAGTGKSTLGRRLLARHEGPAWW 60

************************************************************

ETHR_4284 ATAAPWESRQPYGVVAQMLPDLDVGAGPVAVAERVADRLAASDGTTVAVVDDGHWADEES 120

SACE_4376 ATAAPWESRQPYGVVAQMLPDLDVGAGPVAVAERVADRLAASDGTTVAVVDDGHWADEES 120

************************************************************

ETHR_4284 LQALASVVRHHPRARLLVVAFAVTGDPHASAATLELLPRIATAEIRLSPLTAAQVNELAA 180

SACE_4376 LQALASVVRHHPRARLLVVAFAVTGDPHASAATLELLPRIATAEIRLSPLTAAQVNELAA 180

************************************************************

ETHR_4284 ARGVVLHPSIAERLCHHTQGAARHIAQLLDEVPAATWTRFDPGLPAPAAVAARVRELLAG 240

SACE_4376 ARGVVLHPSIAERLCHHTQGAARHIAQLLDEVPAATWTRFDPGLPAPAAVAARVRELLAG 240

************************************************************

ETHR_4284 CSPTVRAFVEATAVLGPGTAVRDAALLAGVDDGLLTVLDEACATGLVRLAPRGLAEVGPA 300

SACE_4376 CSPTVRAFVEATAVLGPGTAVRDAALLAGVDDGLLTVLDEACATGLVRLAPRGLAEVGPA 300

************************************************************

ETHR_4284 DPMVRAAVLAALAPGRRSGLHRRAADLVDDPARKLRLLVAASPVPDPAVADRLDALATER 360

SACE_4376 DPMVRAAVLAALAPGRRSGLHRRAADLVDDPARKLRLLVAASPVPDPAVADRLDALATER 360

************************************************************

ETHR_4284 ASEGAWAEAASLLIDASRLTEDRLERESRISRAVDALIGAGDVSAAAALVPEVESLRETP 420

SACE_4376 ASEGAWAEAASLLIDASRLTEDRLERESRISRAVDALIGAGDVSAAAALVPEVESLRETP 420

************************************************************

ETHR_4284 LRNAVLGYLAVVRGRVAEAESRLGRAWSLVNAERDPDVAALICQRYVLHALVRCRAGELV 480

SACE_4376 LRNAVLGYLAVVRGRVAEAESRLGRAWSLVNAERDPDVAALICQRYVLHALVRCRAGELV 480

************************************************************

ETHR_4284 TWADRAISLAGPDAPAAVEAAAIRGLGLAATGRAGQARRDYARLGG-------------- 526

SACE_4376 TWADRAISLAGPDAPAAVEAAAIRGLGLAATGRAGQARRDYARLGERVRHGAQAQRVMMA 540

*********************************************

ETHR_4284 ------------------------------------------------------------

SACE_4376 RGWLDLLTDAVDDARAELESAVPTTFLGGSTRISLWARAWLARAQLLAGAWDDAVRTVRD 600

ETHR_4284 ------------------------------------------AGP--------------- 529

SACE_4376 AAPLLERSGIVLTGPLLHWTEVTVHALRGEWEQAEQALLRCDAGPQDYEIMRVPTYLARA 660

***

ETHR_4284 ----ARSAGP-------------AG----------------------------------- 537

SACE_4376 QVAEARADSAGVLRALHPLTQSWAGGGIDEPGHWPWHDMYAHALVLEGRHEEADEFLLPH 720

**: .. **

ETHR_4284 -------DDGPRLARPAHRRGRRR------------------------------------ 554

SACE_4376 ERLAATREHASARARLAAARGRLLGAQGDPAAATTAFEQALELLEGLPLRYDHARVAFAY 780

:... ** * ***

ETHR_4284 ---TRRAGERR-----------------ADHLPRWFDPDLAVG-----------AGLART 583

SACE_4376 GQTLRRAGKRREADAVLTTARDGFAAMGAATYVERCDRELKAGGVHTLRAERAFNELTPQ 840

****:** * . * :* .* *:

ETHR_4284 RAAPRRGVGRRGAHR---ARRGASARTQRHRPHRSAAALDRGDRARVAR----------- 629

SACE_4376 EEAVARLVARGLSNREAAAELFLSTKTVQYHLTRIYTKLGIRSRAELAALRSASEDPNRE 900

. * * *.* ::* *. *::* ::: * : *. .**.:*

7、Ref: 2; change: E3; domain: No

SeqA Name Len(aa) SeqB Name Len(aa) Score

===========================================================

1 ETHR_0099 220 2 SACE_0101 220 99

1 ETHR_0099 220 3 SCLAV_1358 220 80

1 ETHR_0099 220 4 Amir_4935 221 72

2 SACE_0101 220 3 SCLAV_1358 220 81

2 SACE_0101 220 4 Amir_4935 221 72

3 SCLAV_1358 220 4 Amir_4935 221 74

===========================================================

ETHR_0099 MSKILIAEDEARIAAFIEKGLRANGFTTTVVGDGDTALDYVLTGDFDLVVLDLGLPGKDG 60

SACE_0101 MSKILIAEDEARIAAFIEKGLRANGFTTTVVGDGDTALDYVLTGDFDLVVLDLGLPGKDG 60

SCLAV_1358 MNRILIVEDEERIASFVEKGLRANGFTTTVVGDGDSAFDYAVTGGFDLIVLDIGLPGRDG 60

Amir_4935 MARILIAEDEERIASFVRKGLTANGFATTVVGDGEAALGYALGGGFDLVLLDIGLPVLDG 60

* :***.*** ***:*:.*** ****:*******::*:.*.: *.***::**:*** **

ETHR_0099 FAVLRALRAQRVTVPVIILTARDSVHDTVAGLEGGADDYMTKPFRFEELLARVRLRLRPT 120

SACE_0101 FAVLRALRAQRVTVPVIILTARDSVHDTVAGLEGGADDYMTKPFRFEELLARVRLRLRPT 120

SCLAV_1358 FTVLRQLREARVSVPVIILTARDSVRDTVAGLEGGADDWMTKPFRFEELLARVRLRLRTA 120

Amir_4935 FAVLARLRERGCATPVIILTAQDSVRATVAGLEGGADDYMTKPFRFEELLARVRLRLRSP 120

*:** ** :.*******:***: ***********:*******************..

ETHR_0099 DRAPKVTVLRDGELSLDLRTRRAQVPEGTVDLTAREFSMLELFLRHSGQVLSREQILSHV 180

SACE_0101 DRAPEVTVLRDGELSLDLRTRRAQVPEGTVDLTAREFSMLELFLRHSGQVLSREQILSHV 180

SCLAV_1358 ARAPEVTVLRNGELSLDLRTRRARSGERTVDLTAREFVLLELFLRHPGQVLSREQILSHV 180

Amir_4935 EAVVERTVLRAGGLALDLRTRRVDVDGVAVDLTAREFALLELLLRHHRRVLSREQILSHV 180

. : **** * *:*******. :******** :***:*** :***********

ETHR_0099 WGYDFDPGSNVVDVYVRALRRKIGSTRIHTVRGMGYRLGV- 220

SACE_0101 WGYDFDPGSNVVDVYVRALRRKIGSTRIHTVRGMGYRLGV- 220

SCLAV_1358 WGYDFDPGSNIVDVYVRALRKKLGAGRVETVRGMGYRLPD- 220

Amir_4935 WGYDFDPGSNIVDVYVRALRRKIGAGRIRTARGMGYSFDAG 221

**********:*********:*:*: *:.*.***** :

8、Ref: 1; change: E3; domain: No

SeqA Name Len(aa) SeqB Name Len(aa) Score

=========================================================

1 ETHR_1005 163 2 SACE_1040 163 99

1 ETHR_1005 163 3 Amir_0800 167 60

2 SACE_1040 163 3 Amir_0800 167 60

=========================================================

ETHR_1005 ---------------MTETG-LDRLPTRTELLAYRSFLRAHARVTCCLEGDLIAEQRLTL 44

SACE_1040 ---------------MTETG-LDRLPTRTELLAYRSFLRAHARVTRCLEGDLIAEQRLTL 44

Amir_0800 MLFLPAPTLSVTSAPVSDTGPTRRAPTRDELVVWRSFLRAHARLTRTLEAELVLDQRLTL 60

:::** * *** **:.:*********:* **.:*: :*****

ETHR_1005 AAYDVLEALTEAPEQRLRMTELADAVLLSRSGVTRLVDRLERLGLVRRVRVDTDGRGVQA 104

SACE_1040 AAYDVLEALTEAPEQRLRMTELADAVLLSRSGVTRLVDRLERLGLVRRVRVDTDGRGVQA 104

Amir_0800 ASYDVLVQLAERPDRRMRMTELADAVLLSRSGVTRLVDRLERSGLVLRERVDGDGRGVVA 120

*:**** *:* *::*:************************* *** * *** ***** *

ETHR_1005 VITERGEHRLRTASATHRNGVARYFLSAAEGGELAELTRWCERLADGGVPAPPGQRAQA 163

SACE_1040 VITERGEHRLRTASATHRNGVARYFLSAAEGGELAELTRWCERLADGGVPAPPGQRAQA 163

Amir_0800 VLTEAGLDRLRGASGTHLRGVARHFADALDGEDLAEFRRICEKLAEG------------ 167

*:** * .*** **.** .****:* .* :* :***: * **:**:*

9、Ref: 1; change: E3; domain: No

SeqA Name Len(aa) SeqB Name Len(aa) Score

===================================================================

1 ETHR_2283 619 2 SACE_2347 619 99

1 ETHR_2283 619 3 AAM97369[RubS] 661 30

2 SACE_2347 619 3 AAM97369[RubS] 661 30

===================================================================

CLUSTAL 2.0.12 multiple sequence alignment

ETHR_2283 -------------MTLRINVIGPLEVVCRDEVVTPSQPELRRLLALLAIEAGSVVRTETI 47

SACE_2347 -------------MTLRINVIGPLEVVCRDEVVTPSQPKLRRLLALLAIEAGSVVRTETI 47

AAM97369 MAATHTGGSLRGNAVIEIGLLGSMRIRRNGEDVTPSAPKLRQVLALLVLNANSLVSVDQL 60

.:.*.::*.:.: ..* **** *:**::****.::*.*:* .: :

ETHR_2283 TRELWGSQPTGKLARTVQTYVSHLRRVLAPAG-----------PACCTLTHVARVGYRLR 96

SACE_2347 TRELWGSQPTGKLARTVQTYVSHLRRVLAPAG-----------PACCTLTHVARVGYRLR 96

AAM97369 CEELWEDHPPLSALTTLQTYIYQLRRRLLLATGQHGAAFGSRPPHGCPAILTRVGGYELR 120

.*** .:*. . *:***: :*** * * * *. . **.**

ETHR_2283 MAEGARVDLDRLLTLRDSAEAGTGGDAPDTDAVDILRDAVSLCRGEVLSDVSLGPVLQQH 156

SACE_2347 MAEGARVDLDRLLTLRDSAEAGTGGDAPDTDAVDILRDAVSLCRGEVLSDVSLGPVLQQH 156

AAM97369 LDDKQSVDAYRFDQLIEQGMAQLRTGAGEEEAARTLKAALSLWQGGALVDVSTGRRLSAW 180

: : ** *: * :.. * .* : :*. *: *:** :* .* *** * *.

ETHR_2283 RSRIESVRVGVLDRYLRACLRLERPHAVLEQADRIMREDSGQEELYTSLLLAFAAAGRHS 216

SACE_2347 RSRIESVRVGVLDRYLRACLRLERPHAVLEQADRIMREDSGQEELYTSLLLAFAAAGRHS 216

AAM97369 STQLEERRKSVLEQRFSLELQLGRHHTVLDELSEAFRAHPTHEAFAGQLMRALHRCGRRP 240

:::*. * .**:: : *:* * *:**:: .. :* .. :* : .*: *: .**:.

ETHR_2283 EGAEVFQRLRREHLERTGAEPGAAVRDAYRELVAGTREPSVPERTASLAAPAPPPAAVAV 276

SACE_2347 EGAEVFQRLRREHLERTGAEPGAAVRDAYRELVAGTREPSVPERTASLAAPAPPPAAVAV 276

AAM97369 DALNTFRTLRSHLVEELGLEPSVQLQRLHQEVLADRGHLRGPAPAKAETVSGGR-RLVPX 299

:. :.*: ** . :*. * **.. :: ::*::*. . * : : :... *.

ETHR_2283 PRQLPLDVPAFTGLARQLRIAETALTTGGAQP-PATVAVVGSPGSGKSAFCIRLANRVAH 335

SACE_2347 PRQLPLDVPAFTGLARQLRIAETALTTGGAQP-PATVAVVGSPGSGKSAFCIRLANRVAH 335

AAM97369 PAQLPADVGDFVGRERELDQLETFLGADRPSTGMRVVEVHGPPGVGKSAFAVRAAHRLRP 359

* *** ** *.* *:* ** * :. ... .* * *.** *****.:* *:*:

ETHR_2283 LFPDGQLHADLAGVTP-SQALAGFLRALRTGARIPENP-----HERARLFRECTADSRLL 389

SACE_2347 LFPDGQLHADLAGVTP-SQALAGFLRALRTGARIPENP-----HERARLFRECTADSRLL 389

AAM97369 RFPDGQLFIDLSAVGEGSQQLADVLSACLTACGIQREKQPSGLGELSRLFRTWTADRRVL 419

******. **:.* ** **..* * *.. * .: * :**** *** *:*

ETHR_2283 VVLDNATGRTDVSLLRPGSPRSAVLIACASRHPAALAGTTVELPRMGVRELLEVFADRAG 449

SACE_2347 VVLDNATGRTDVSLLRPGSPRSAVLIACASRHPAALAGTTVELPRMGVRELLEVFADRAG 449

AAM97369 VVVDDALTASQIRAVLPGGSGCAVIATNRYRAHSLSTGRKIVLPALSPEESLQLYDRVAG 479

**:*:* ::: : **.. .**: : * : :* .: ** :. .* *::: **

ETHR_2283 RTRVEREPDEARALVAECSGLPLAVTSLAALLRRRPHWSVGRLLGRVREDR-----FALG 504

SACE_2347 RTRVEREPDEARALVAECSGLPLAVTSLAALLRRRPHWSVGRLLGRVREDR-----FALG 504

AAM97369 ERSRQEDPGAVQELIGLCEGLPLAIRAVAGRLTARPGWSAARLASRLRGNHRMLLELPAG 539

. :.:*. .: *:. *.*****: ::*. * ** **..** .*:* :: :. *

ETHR_2283 DDDLLASVRRALAALGDTDRAGLSELVRKAPAQESLSVRWAAGALGVPVRDAERVLEHLV 564

SACE_2347 DDDLLASVRRALAALGDTDRAGLSELVRKAPAQESLSVRWAAGALGVPVRDAERVLEHLV 564

AAM97369 TQSLMTTVAASHRHLPARSRELLRLLLQRERPRWSLDEVVTR--LRPGSGDAETLLEYLV 597

:.*:::* : * .* * *::: .: **. : * *** :**:**

ETHR_2283 EHRLADPVAT-DPAAASSRYRIDPLYRLVVRELDAR---------GPGPLRSSEATEQLR 614

SACE_2347 EHRLADPVAT-DPAAASSRYRIDPLYRLVVRELDAR---------GPGPLRSSEATEQLR 614

AAM97369 DVHLVQEHLTPDAELLYSVPRLTRQALILLSRGTPAGDVPNGRMVGAGVVAGTGVTLMKA 657

: :*.: * *. * *: ::: . . *.* : .: .*

ETHR_2283 TIRRR 619

SACE_2347 TIRRR 619

AAM97369 DVRR- 661

:**

10、Ref: No; change: uncertain; domain: No

SeqA Name Len(aa) SeqB Name Len(aa) Score

=========================================================

1 ETHR_2842 918 2 SACE_2927 918 99

=========================================================

ETHR_2842 MGPAELLRADGSTAGLGAAKRRSVLAALALDLNRVVSMDRLLDVVWEGSPPLSAKAALQG 60

SACE_2927 MGPAELLRADGSTAGLGAAKRRSVLAALALDLNRVVSMDRLLDVVWEGSPPPSAKAALQG 60

*************************************************** ********

ETHR_2842 HIAQLRKVLGDGVELVTRSPGYQLVADRSQLDVTRFEDLLAEARTATDAEAVELLRTALA 120

SACE_2927 HIAQLRKVLGDGVELVTRSPGYQLVADRSQLDVTRFEDLLAEARTATDAEAVELLRTALA 120

************************************************************

ETHR_2842 LRRGPVLADVPAERLRRVISARMEESVVTAIQELGRRLHRLGRTAEGIDLLHEAVALRPL 180

SACE_2927 LRRGPVLADVPAERLRRVISARMEESVVTAIQELGRRLHRLGRTAEGIDLLHEAVALRPL 180

************************************************************

ETHR_2842 REPLVELLVLSLHHAGRQAEALNVYHDTRTRLADELGVDPGIGLRQAFHTVLTAHDAPEP 240

SACE_2927 REPLVELLVLSLHHAGRQAEALNVYHDTRTRLADELGVDPGIGLRQAFHTVLTAHDAPEP 240

************************************************************

ETHR_2842 TAPKCVPLQLPRENRGFAGREAELAKLGPNGGADGAIRILVGPAGVGKTALALRWAHQVA 300

SACE_2927 TAPKCVPLQLPRENRGFAGREAELAKLGPNGGADGAIRILVGPAGVGKTALALRWAHQVA 300

************************************************************

ETHR_2842 ARFPDGHLFADLRGFDETDPVAPDHVLTGFLRALGVPDARIPADADERAALYRSAVAGRR 360

SACE_2927 ARFPDGHLFADLRGFDETDPVAPDHVLTGFLRALGVPDARIPADADERAALYRSAVAGRR 360

************************************************************

ETHR_2842 MLVVLDNARSAAQVRPLLPGTSSCAVLVSSRSRLDDLAATEGAVRVTVPALSRDEAVTVL 420

SACE_2927 MLVVLDNARSAAQVRPLLPGTSSCAVLVSSRSRLDDLAATEGAVRVTVPALSRDEAVTVL 420

************************************************************

ETHR_2842 GLVLGADRVAAEPAAAAELAELCDRLPLALRIAAARGSSHQHGTLRAMVEAFSDERHRLH 480

SACE_2927 GLVLGADRVAAEPAAAAELAELCDRLPLALRIAAARGSSHQHGTLRAMVEAFSDERHRLH 480

************************************************************

ETHR_2842 RLSLPDSGSTVRTALAWSYRRLDAASARLFRRLGEHPGTDVDRGAAAALAGTTVAEVQPR 540

SACE_2927 RLSLPDSGSTVRTALAWSYRRLDAASARLFRRLGEHPGTDVDRGAAAALAGTTVAEVQPR 540

************************************************************

ETHR_2842 LESLISVHLLHKSGPERYARHDLVRLYTAAVAEEEPAADRHAATERLLDYYLHTADAGRR 600

SACE_2927 LESLISVHLLHKSGPERYARHDLVRLYTAAVAEEEPAADRHAATERLLDYYLHTADAGRR 600

************************************************************

ETHR_2842 LVTDEAWQPAMRVARPPAESPELSTVKEALDWFRAEETNLHRALALAGARGDHGRAWRLA 660

SACE_2927 LVTDEAWQPAMRVARPPAESPELSTVKEALDWFRAEETNLHRALALAGARGDHGRAWRLA 660

************************************************************

ETHR_2842 LCLERFQHHLGDLPAQAEAARLGLAAARELGDNQAQAVFHVRVGENLVRSGRAGDAVAHG 720

SACE_2927 LCLERFQHHLGDLPAQAEAARLGLAAARELGDNQAQAVFHVRVGENLVRSGRAGDAVAHG 720

************************************************************

ETHR_2842 EQAVRLGRDEPQDACNAMLGLGRFLHAAGRPAEALARLTGSIEVAGAVGNVAVETYALLG 780

SACE_2927 EQAVRLGRDEPQDACNAMLGLGRFLHAAGRPAEALARLTGSIEVAGAVGNVAVETYALLG 780

************************************************************

ETHR_2842 KAWVHQSEGERTDAMDALRRSVELVRDNGSRVHGAAVLLIAGELVREFGEHDKALRMFSR 840

SACE_2927 KAWVHQSEGERTDAMDALRRSVELVRDNGSRVHGAAVLLIAGELVREFGEHDKALRMFSR 840

************************************************************

ETHR_2842 GLSAARDAEDLVFQARHHRAIGNTLAHLGSRAAAVPHWTRAERLRAALGLPDGGDVTLNG 900

SACE_2927 GLSAARDAEDLVFQARHHRAIGNTLAHLGSRAAAVPHWTRAERLRAALGLPDGGDVTLNG 900

************************************************************

ETHR_2842 SHGHRAPHPHGFPELACT 918

SACE_2927 SHGHRAPHPHGFPELACT 918

******************

11、Ref: 2; change: uncertain; domain: No

SeqA Name Len(aa) SeqB Name Len(aa) Score

=========================================================

1 ETHR_3260 96 2 SACE_3348 96 98

1 ETHR_3260 96 3 Amir_5395 282 48

1 ETHR_3260 96 4 Snas_4247 410 37

2 SACE_3348 96 3 Amir_5395 282 48

2 SACE_3348 96 4 Snas_4247 410 38

3 Amir_5395 282 4 Snas_4247 410 34

=========================================================

ETHR_3260 ------------------------------------------------------------

SACE_3348 ------------------------------------------------------------

Amir_5395 ----------------------------------------------MPGNAYTSVKSRTV 14

Snas_4247 MVTSHCDVYDLRSVTSCHTESRNAMCVFRLGVVRFSYCELMRHNERMTARKSLTVRKRRL 60

ETHR_3260 ------------------------------------------------------------

SACE_3348 ------------------------------------------------------------

Amir_5395 AGALRTYREQHGLSCEDVASVLGVSSSKISRMETGKSGLQVEDVSALLGYYKVPGARRRE 74

Snas_4247 VRALRQLRKDSGITLEKAAEHLDINHTSLSRIETGVAAVKLPYVESLLRLYGVPEARQEE 120

ETHR_3260 ------------------------------------------------------------

SACE_3348 ------------------------------------------------------------

Amir_5395 LLDLMRRGEELGWWER-QAGLPKLWRALIDFENKATGVHNYESMVVPGLVQTAEYTRALI 133

Snas_4247 LLQLTREAKQRGWWQAYKDILSSEYADFIGFETEANETRTYELDTVPGLLETEDYARALI 180

ETHR_3260 ----------------------------------------------------MKRQLQHL 8

SACE_3348 ----------------------------------------------------MKRQLQHL 8

Amir_5395 RSLDPALPEHELDALVTTRMARQAVLNRASAPEYLAVLHEAALRIRVGDEGVMRRQLRYL 193

Snas_4247 SAQLPGATAEDIEKRVKLRASRQDRLKEDPKLSVWAILGEAALRYQVGGMKVLRAQLEYL 240

:: **.:*

ETHR_3260 LTVSEASNVTMRVVPRSAGAYDGLYGAFLLLEYQEEPDIVFVENHVTALFLAEEADLASY 68

SACE_3348 LTVSEASNVTMRVVPRSAGAYDGLYGAFLLLEYQEEPDIVFVENHVTALFLEEEADLASY 68

Amir_5395 LDVAERSNVVVRVTPMGAGAHVGLSGAFTLLEFAHEPAVVFVENQSTGLFLDGAAEVDGY 253

Snas_4247 LQLQREPNITIQVLPFSAGAHPGMAGPFVILGFDDDPDIVYLEGLTSALYLEDLGELERY 300

* : . .*:.::* * .***: *: *.* :* : .:* :*::*. :.*:* .:: *

ETHR_3260 RMALRNILGSALAPAATRDLISSVAAEH-------------------------------- 96

SACE_3348 RMALRNILGSALAPAATRDLISSVAAEH-------------------------------- 96

Amir_5395 RRAWGRIVDVSLSPGATAELLAELVEERP------------------------------- 282

Snas_4247 KMVFERLLAEALSPAASDRLIREASKELCHFANNGRRRGMAAQGDDLARARWRKGRRTQA 360

: . .:: :*:*.*: *: . *

ETHR_3260 --------------------------------------------------

SACE_3348 --------------------------------------------------

Amir_5395 --------------------------------------------------

Snas_4247 NGNCVEVALVESVYVRDSKLDTTGTFPTLSVSSTEWKNFLLAIANNDKTG 410

12、Ref: 1; change: E3; domain: Yes

SeqA Name Len(aa) SeqB Name Len(aa) Score

=========================================================

1 ETHR_3703 388 2 SACE_3795 388 99

1 ETHR_3703 388 3 Amir_2145 346 85

2 SACE_3795 388 3 Amir_2145 346 86

=========================================================

ETHR_3703 MTEPGNGEGGTAGAPARASEQVNTPARDAQLLERTVFEVKRVIVGQDRLVERVLVGLLAK 60

SACE_3795 MTEPGNGEGGTAGAPARASEQVNTPARDAQLLERTVFEVKRVIVGQDRLVERVLVGLLAK 60

Amir_2145 MSEP-------------AAEAHTTPARDAQLLERTVFEVKRVIVGQDRLVERMLVGLLAK 47

*:** *:* .*****************************:*******

ETHR_3703 GHILLEGVPGVAKTLAVETFARVVGGSFSRLQFTPDLVPADILGTRIYRQGSERFDVELG 120

SACE_3795 GHILLEGVPGVAKTLAVETFARVVGGSFSRLQFTPDLVPADILGTRIYRQGSERFDVELG 120

Amir_2145 GHLLLEGVPGVAKTLAVETFATVVGGSFSRVQFTPDLVPADILGTRIYRQASESFDVELG 107

**:****************** ********:*******************.** ******

ETHR_3703 PVLANFVLADEIDRAPAKVQSALLEVMAERHVSIGGESFPMPSPFLVLATQNPIENEGVY 180

SACE_3795 PVLANFVLADEINRAPAKVQSALLEVMAERHVSIGGESFPMPSPFLVLATQNPIENEGVY 180

Amir_2145 PVVANFVLADEINRAPAKVQSAMLEVMAERHVSIGGKTFPMPTPFLVLATQNPIENEGVY 167

**:*********:*********:*************::****:*****************

ETHR_3703 PLPEAQRDRFLFKLQVEYPSAEEEREIVYRMGVASPEPNPVLDPDELTRLQGVASKVFVH 240

SACE_3795 PLPEAQRDRFLFKLQVEYPSAEEEREIVYRMGVASPEPNPVLDPDELTRLQGVASKVFVH 240

Amir_2145 PLPEAQRDRFLFKIQVEYPTAEEEREIVYRMGVEAPVPQQVLSPEELVRLQGVASRVFVH 227

*************:*****:************* :* *: **.*:**.*******:****

ETHR_3703 HALVDYVVRLVLATRTPNDHGLSDIAGWVSYGASPRASLGIVAAARALALVRGRDYVLPQ 300

SACE_3795 HALVDYVVRLVLATRTPNDHGLSDIAGWVSYGASPRASLGIVAAARALALVRGRDYVLPQ 300

Amir_2145 HALVDYVVRLVIATRAPKEHQLGDVAGWVAYGASPRASLGIIAASRALALVRGRDYVLPQ 287

***********:***:*::* *.*:****:***********:**:***************

ETHR_3703 DVVDVVPDVLRHRLVLSYDALADGIPVDHIVNRVLQTVPLPQVSARPQ-GGPPAPVGAPA 359

SACE_3795 DVVDVVPDVLRHRLVLSYDALADGIPVDHIVNRVLQTVPLPQVSARPQ-GGPPAPVGAPA 359

Amir_2145 DVVDVVPDVLRHRLVLSYDALADGVPLDHIITRVLQTVPLPQVSARPQAGGAPQPVGRP- 346

************************:*:***:.**************** **.* *** *

ETHR_3703 PGPSGPGHAQPPGGGPAYGQGPLGPTQHQ 388

SACE_3795 PGPSGPGHAQPPGGGPAYGQGPLGPTQHQ 388

Amir_2145 -----------------------------

13、Ref: 2; change: ETHR; domain: Yes

SeqA Name Len(aa) SeqB Name Len(aa) Score

=========================================================

1 ETHR_4682 246 2 SACE_4775 246 99

1 ETHR_4682 246 3 Snas_3346 241 66

1 ETHR_4682 246 4 Kfla_4419 247 63

2 SACE_4775 246 3 Snas_3346 241 67

2 SACE_4775 246 4 Kfla_4419 247 63

3 Snas_3346 241 4 Kfla_4419 247 56

=========================================================

ETHR_4682 MAWSTREIAELAGTSLRAVRHYHEVGLLAEPERRANGYKKYGVAHLVRLLRIKRLTDLGF 60

SACE_4775 MAWSTREIAELAGTSLRAVRHYHEVGLLAEPERRANGYKKYGVAHLVRLLRIKRLTDLGF 60

Snas_3346 MSWSTRELAELAGTSLRAVRHYHEVGLLEEPERRTNGYKQYSVRHLVRLLRIKRLVDLGF 60

Kfla_4419 MAWSTREIAQLAGTTLRAVRHYHDIGLLAEPQRRTNGYKQYEVAHLVRILRIKRLTELGF 60

*:*****:*:****:********::*** **:**:****:* * ****:******.:***

ETHR_4682 SLSQIAAMGEADDHPADALRALDAELAATIERLQRVRGELAVILRQAVPTDLPPEFAP-A 119

SACE_4775 SLSQIAAMGEADDHPADALRALDAELAATIERLQRARGELAVILRQAVPTDLPPEFAP-A 119

Snas_3346 TLSQIADMADTDTHPEEALRTLDAELATTIERLQRARAELRFILRHSAPADLPPELTP-I 119

Kfla_4419 SLSQIATMDDTDDQPVEALRTLDAELAATIERLQRARVELGVILRQPTPAELPPGFAASD 120

:***** * ::* :* :***:******:*******.* ** .***:..*::*** ::.

ETHR_4682 AVPELSEADRSFVVVLTRVLGPRGMQAYSEMLQDLPDDPTAPEFGELPADADEPTRQDVA 179

SACE_4775 AVPELSEADRSFVVVLTRVLGPRGMQAYSEMLQDLPDDPTAPEFGELPADADEPTRQDVA 179

Snas_3346 SGHTLSEADRSFVTVMNQTLGPQGRQAYSDMVRSVADDPIAHEFDNLPADADEPTRQDLA 179

Kfla_4419 VVARMSPADRSFIVVLTRVLGAETMRAWADLLREPVIDPVAQRFDTLPADADEQTRAAVA 180

:* *****:.*:.:.**.. :*::::::. ** * .*. ******* ** :*

ETHR_4682 ERLVPYVRALHAEHPGLRDANADAPGGAQFAEQTVGKALVDLYNPAQLDVMRRIESLLRA 239

SACE_4775 ERLVPYVRALHAEHPGLRDANADAPGGAQFAEQTVGKALVDLYNPAQLDVMRRIESLLRA 239

Snas_3346 ERLTPYLRGVYKAHPGLRTITADAPRGPDFATRMTGKALVELYNPAQVDVLARVAPHIAD 239

Kfla_4419 RDLVPYVRTLSAKHPDVKITNAGTRYSARYVEQAVGQALRELYNSAQLDVLRRISRLLSD 240

. *.**:* : **.:: .*.: .. :. : .*:** :***.**:**: *: :

ETHR_4682 SGPTDPT 246

SACE_4775 SGPTDPT 246

Snas_3346 SA----- 241

Kfla_4419 PHDQPAQ 247

.

15、Ref: No; change: Null; domain: No

SeqA Name Len(aa) SeqB Name Len(aa) Score

=========================================================

1 ETHR_4945 245 2 SACE_5046 245 99

=========================================================

CLUSTAL 2.0.12 multiple sequence alignment

ETHR_4945 MNADHRRTEGRHMTIGNADSPPQSRTAWVAERIREDVAAGTIQPGELIKQTVLAKRYGVS 60

SACE_5046 MNADHRRTEGRHMTIGNADSPPQSRTAWVAERIREDVAAGTIQPGELIKQTVLAKRYGVS 60

************************************************************

ETHR_4945 PTPVREALRMLEADGMIVYSTHKGATVREMTPETAADLYRLRAAVESVAAGMAVERMTSE 120

SACE_5046 PTPVREALRMLEADGMIVYSTHKGATVREMTPETAADLYRLRAAVESVAAGMAVERMTSE 120

************************************************************

ETHR_4945 GLQEIERQHAEVDRAVREGAAPAELSRLNKQFHFSIYAQSSPLVLQYVEALWVRFTPPAT 180

SACE_5046 GLQEIERQHAEVIRAVREGAAPAELSRLNKQFHFSIYAQSSPLVLQYVEALWVRFTPPAT 180

************ ***********************************************

ETHR_4945 VWGSEDAAMALQCDHDAILKAVRKGDADAAARLTAEHVQHAAAIRDANPELRAAGGQDRE 240

SACE_5046 VWGSEDAAMALQCDHDAILKAVRKGDADAAARLTAEHVQHAAAIRDANPELRAAGGQDRE 240

************************************************************

ETHR_4945 DHTGL 245

SACE_5046 DHTGL 245

*****

16、Ref: No; change: Null; domain: No

SeqA Name Len(aa) SeqB Name Len(aa) Score

=========================================================

1 ETHR_5286 287 2 SACE_5410 287 99

=========================================================

ETHR_5286 MTQSDPTVRSQELGDELRALREANTLSLVDAARRIDASGSKLSRIETGISAPSAEDVSGL 60

SACE_5410 MTQSDPTVRSQELGDELRALREANTLSLVDAARRIDASGSKLSRIETGISAPSAEDVSGL 60

************************************************************

ETHR_5286 LVLYGVTGEKRRELLALARESERRGWWQRNHPGFAERQRTLVSLEAKADSIVNFEAIVVP 120

SACE_5410 LVLYGVTGEKRRELLALARESERRGWWQRNHPGFAERQRTLVSLEAKADSIVNFEAIVVP 120

************************************************************

ETHR_5286 GLLQTGEYTRAIMQGVGVKFESEIEARMVTRLHRHKVLRRERPPRLLAILDELVLHRRIG 180

SACE_5410 GLLQTGEYTRAIMQGVGVKPESEIEARMVTRLHRHKVLRRERPPRLLAILDELVLHRRIG 180

******************* ****************************************

ETHR_5286 GGDVLRRQLEFLVEASTLPNINVRVVPNDGLAHPGIEGAFTVLRRSGRSPVIFAETLTSC 240

SACE_5410 GGDVLRRQLEFLVEASTLPNINVRVVPNDGLAHPGIEGAFTVLRRSGRSPVIFAETLTSC 240

************************************************************

ETHR_5286 IFLEDRIEIERYESALRTLSERALDERQSVQLMTDLARRLDSEATGT 287

SACE_5410 IFLEDRIEIERYESALRTLSERALDERQSVQLMTDLARRLDSEATGT 287

***********************************************

17、Ref: No; change: Null; domain: No

SeqA Name Len(aa) SeqB Name Len(aa) Score

=========================================================

1 ETHR_5435 258 2 SACE_5610 258 99

=========================================================

ETHR_5435 MLRHYDAIGLLRPAHVDAASGYRRYEAAQLCRLNRIIALKDLGFTLQQVGDLLDADVSAE 60

SACE_5610 MLRHYDAIGLLRPAHVDAASGYRRYEAAQLCRLNRIIALKDLGFTLQQVGDLLDADVSAE 60

************************************************************

ETHR_5435 QMRGMLRLRLSELEAAVAADTARLRQVEARLRTIESEGRMPTEDVVVKSIPAVRVAELTA 120

SACE_5610 QMRGMLRLRLSELEAAVAADTARLRQVEARLRTIESEGRMPTEDVVVKSIPAVRVAELTA 120

************************************************************

ETHR_5435 IAGSFGPEDIGPVIRPLYGELCRRMAAAGVSGSGPNIAYYEESPEGDDEVVVHAGVTVAV 180

SACE_5610 IAGSFGPEDIGPVIRPLYGELCRRMAAAGVSGSGPNIAYYEESPEGDDEVVVHAGVTVAV 180

************************************************************

ETHR_5435 EPHAGRGFDVVDLPAVERAATIVHRGDMDEFMPTVQQLAHWIDANGFRAVGLGREHYLSC 240

SACE_5610 EPHAGRGFDVVDLPAVERAATIVHRGDMDEVMPTVQQLAHWIDANGFRAVGLGREHYLSC 240

******************************.*****************************

ETHR_5435 EGGPDQWVTEIQQPITKA 258

SACE_5610 EGGPDQWVTEIQQPITKA 258

******************

18、Ref: No; change: Null; domain: No

SeqA Name Len(aa) SeqB Name Len(aa) Score

=========================================================

1 ETHR_5562 282 2 SACE_5739 282 99

=========================================================

ETHR_5562 MQQRQLGNELRKLREAAGITQEVASEHLGKAHNKISRVETAKVGISGLELEALLSLYKAS 60

SACE_5739 MQQRQLGNELRKLREAAGITQEVASEHLGKAHNKISRVETAKVGISGLELEALLSLYKAS 60

************************************************************

ETHR_5562 PKDKVWCRELAKGARRRRGRPKEATLYRGPRWFRAFRDFEQSATEVMMVGSEVLPGILQT 120

SACE_5739 PKDKVWCRELAKGARRRRGRPKEATLYRGPRWFRAFRDFEQSATEVMMVGSEVLPGILQT 120

************************************************************

ETHR_5562 EEYTRSIFAGRGDDPNGKDVEDHVRIRKERQELLTREEASHFSFVLSESALRRQIGDPAT 180

SACE_5739 EEYTRSIFAGRGDDPNGKDVEDHVRIRKERQELLTREEASHFSFVLSESALRRQIGDPAT 180

************************************************************

ETHR_5562 MAEQLDYLAEVALLTNINIQVIPFDTLSYDAVGSDFVIFRFDDDTSTDIVYIEIYGDAIY 240

SACE_5739 MAEQLDYLAEVALLTNINIQVIPFDKLSYDAVGSDFVIFRFDDDTSTDIVYIEIYGDAIY 240

*************************.**********************************

ETHR_5562 IDKPAEAVRRYNELLSRLYGIALGPVESRNFIRELASQLAGR 282

SACE_5739 IDKPAEAVRRYNELLSRLYGIALGPVESRNFIRELASQLAGR 282

******************************************
